# Cofilin-mediated Neuronal Apoptosis via p53 Translocation and PLD1 Regulation

**DOI:** 10.1038/s41598-017-09996-3

**Published:** 2017-09-14

**Authors:** Tian Liu, Fang Wang, Patrick LePochat, Jung-A. A. Woo, Mohammed Zaheen Bukhari, Kyung Woo Hong, Courtney Trotter, David E. Kang

**Affiliations:** 1 USF Health Byrd Alzheimer’s Institute, Department of Molecular of Medicine, University of South Florida, Morsani College of Medicine, Tampa, FL 33613 USA; 2 James A. Haley Veteran’s Administration Hospital, Tampa, FL 33612 USA

## Abstract

Amyloid β (Aβ) accumulation is an early event in the pathogenesis of Alzheimer’s disease (AD), leading to mitochondrial and synaptic dysfunction, tau accumulation, and eventual neuronal death. While the p53 apoptotic pathway has clearly been associated with Aβ deposits and neuronal apoptosis, the critical upstream factors contributing to p53 activation in AD are not well understood. We have previously shown that cofilin activation plays a pivotal role in Aβ-induced mitochondrial and synaptic dysfunction. In this study, we show that activated cofilin (S3A) preferentially forms a complex with p53 and promotes its mitochondrial and nuclear localization, resulting in transcription of p53-responsive genes and promotion of apoptosis. Conversely, reduction of endogenous cofilin by knockdown or genetic deficiency inhibits mitochondrial and nuclear translocation of p53 in cultured cells and in APP/PS1 mice. This cofilin-p53 pro-apoptotic pathway is subject to negative regulation by PLD1 thorough cofilin inactivation and inhibition of cofilin/p53 complex formation. Finally, activated cofilin is unable to induce apoptosis in cells genetically lacking *p53*. These findings taken together indicate that cofilin coopts and requires the nuclear and mitochondrial pro-apoptotic p53 program to induce and execute apoptosis, while PLD1 functions in a regulatory multi-brake capacity in this pathway.

## Introduction

Alzheimer’s disease (AD) is a neurodegenerative condition in which amyloid β (Aβ) peptide accumulation and hyperphosphorylated tau aggregation are the major pathological hallmarks. Specifically, Aβ accumulation contributes to neurodegeneration in part via activating apoptotic proteins to induce mitochondrial dysfunction. Our previous study showed that Aβ-induced mitochondrial dysfunction is mediated by Slingshot-1 homolog (SSH1)-dependent activation of cofilin, which leads to the translocation of cofilin to mitochondria^1, 2^. As a member of a family of actin-binding protein, cofilin plays an essential role in the cytoskeletal dynamics and signaling via its F-actin severing activity. Increasing evidence shows that cofilin is involved in the neurotoxicity processes observed in neurodegenerative diseases, including AD^2, 3^, Parkinson disease (PD)^4^ and Huntington disease (HD)^5^. The activity of cofilin depends on its phosphorylation status, as LIM kinase-1 (LIMK1)-dependent phosphorylation and SSH1-dependent dephosphorylation of cofilin on Ser-3 inactivates and activates its F-actin severing activity, respectively^6–8^. While current studies indicate that the active dephosphorylated cofilin exerts pro-apoptotic activity via its ability to translocate to mitochondria^9, 10^, other mechanisms that regulate or mediate cofilin-induced neurotoxicity are completely unknown.

P53, a well-characterized tumor suppressor protein, can induce cell death both by extrinsic and intrinsic apoptotic pathways^11^. A number of studies have shown that p53 gains or loses its activity depending on its localization^12^. On the one hand, p53 can translocate from the cytosol to the nucleus and induces the transcription of its pro-apoptotic target genes, including bax, p21 and puma^13^. On the other hand, p53 can directly translocate to mitochondria and induce mitochondrial apoptosis in a transcription-independent manner^14, 15^. Interestingly, the process of p53 trafficking is associated with cytoskeletal proteins including actin and microtubules. Some studies show that wild type p53 induces reorganization of the actin cytoskeleton and modulates various cytoskeleton associated proteins^16, 17^. Other studies indicate that p53 trafficking is regulated by actin, microtubules, and other cytoskeletal proteins^18–21^. Interestingly, it has been shown that F-actin polymerization is associated with retention of cytoplasmic p53, thus impairing nuclear import^18^. In brains of AD patients and APP transgenic mouse models, oligomeric Aβ (100 μΜ) deposition is associated with p53 accumulation in neurons undergoing apoptosis *in vivo* ^22, 23^, and oligomeric Aβ (1.5 μΜ) treatment has been shown to activate p53, driving the transcription of its nuclear targets (i.e. PUMA & Foxo3a) and promoting apoptosis *in vitro* ^23^.

Phospholipase D 1 (PLD1), an enzyme of the phospholipase superfamily, catalyzes the hydrolysis of phosphatidylcholine (PC) into phosphatidic acid (PA) and choline in response to various stimuli. PLD1 is involved in a large variety of early and late cellular responses, including superoxide production, endocytosis, exocytosis, vesicle trafficking, glucose transport, mitogenesis and apoptosis^24^. PLD1 is also involved in actin dynamics and is activated via its interaction with phosphorylated cofilin upon extracellular stimulation by carbachol^25^. Meanwhile, PLD1 also possesses a protective role against cellular apoptosis by enhancing the turnover of p53^26^. In this study, we report on a novel tripartite molecular link between cofilin and p53-mediated apoptotic pathways and show the upstream and downstream interdependence of cofilin and p53 for the induction and execution of mitochondrial dysfunction and apoptosis, while PLD1 functions as a regulatory multi-brake system in this pathway.

## Results

### Activation of PLD1 attenuates Cofilin-S3A-induced mitochondrial dysfunction and cell death

**Figure 1:**
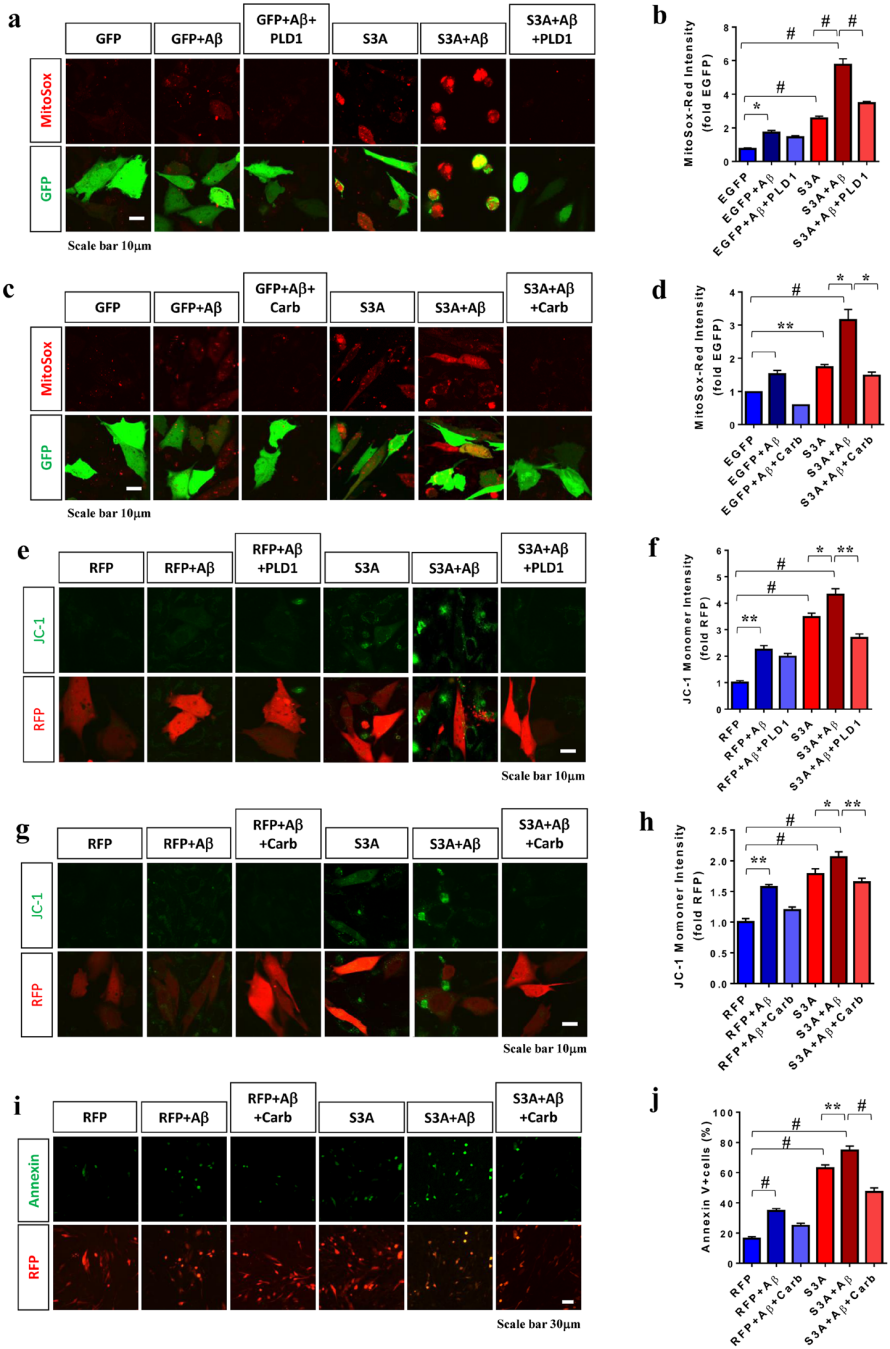
Activation of PLD1 attenuates Cofilin-S3A-induced mitochondrial stress and cell death. (**a**–**d**) Hippocampus-derived HT22 neuroblastoma cells co-transfected with EGFP or Cofilin-S3A-EGFP with/without PLD1 for 24 h, treated with/without Aβ1-42 oligomers (1 μM) and/or carbachol (1 mM) for 24 h, and subjected to MitoSox-Red staining followed by confocal imaging. (**a,c**). Representative images of MitoSox-Red and GFP fluorescence shown. (**b,d**) Quantitative graphs showing MitoSox-Red intensity normalized to EGFP control (1-way ANOVA, post hoc Tukey, ^*^P < 0.05, ^#^P < 0.0005, n = 6 replicates). (**e**–**h**) HT22 cells co-transfected with mRFP or Cofilin-S3A-mRFP with/without PLD1 for 24 h, treated with/without Aβ1-42 oligomers (1 μM) and/or carbachol (1 mM) for 24 h, and subjected to JC-1 staining followed by confocal imaging. (**e,g**). Representative images of JC-1 and mRFP fluorescence shown. (**f,h**) Quantitative graphs showing the JC-1 monomer (green) intensity normalized to mRFP control (1-way ANOVA, post hoc Tukey, ^*^P < 0.05, ^**^P < 0.005, ^#^P < 0.0005, n = 6 replicates). (**i,j**) HT22 cells transfected with mRFP or Cofilin-S3A-mRFP for 24 h, treated with Aβ1-42 oligomers (1 μM) and/or carbachol (1 mM) for 24 h, and subjected to Annexin-V staining followed by confocal imaging. (**i**) Representative images of Annexin V and mRFP fluorescence shown. (**j**) Quantitative graph showing the percentage of Annexin V+ among mRFP+ cells (1-way ANOVA, post hoc Tukey, ^*^P < 0.05, ^**^P < 0.005, ^#^P < 0.0005, n = 6 replicates). All error bars represent S.E.M.

Multiple studies have shown that PLD1 exerts a protective role in programmed cell death (PCD)^27, 28^ and that its activity is induced by carbachol^25, 29^. Moreover, it has been shown that PLD1 binds to and is activated by phosphorylated cofilin (e.g. upon extracellular stimulation with carbachol)^25^. In our initial studies, we found that the non-phosphorylable and constitutively active form of cofilin (S3A cofilin) promoted cell death, while the phospho-mimic S3E cofilin protected against oligomeric Aβ42_O_-induced cell death (Supplemental Fig. 1). Therefore, we assessed whether PLD1 can protect against cofilin and Aβ42_O_-induced cell death. The mouse hippocampus-derived HT22 neuroblastoma cells have previously been characterized for oligomeric Aβ42-induced neurotoxicity, mitochondrial dysfunction, and apoptosis^1^. We transfected HT22 with control vector (mRFP or EGFP) or cofilin-S3A (mRFP or EGFP fusion variants) with or without PLD1, followed by treatment with or without 1 μM Aβ42_O_ and/or carbachol (1 mM) for 24 hours. Then live cells were subjected to measurement of mitochondrial superoxide levels (Mitosox-Red), mitochondrial membrane potential (Δψm, JC-1), and cell death (Annexin V). Compared to EGFP transfected cells, cofilin-S3A-EGFP transfected cells exhibited a significant increase in Mitosox-Red (Fig. 1a–d) and JC-1 monomer intensity (Fig. 1e–h), indicative of mitochondrial superoxide overproduction and reduction in Δψm, respectively. We were unable to assess JC-1 oligomer intensity due to its spectral overlap with mRFP. As expected from reduced mitochondrial health in cofilin-S3A transfected cells, cofilin-S3A transfected cells also exhibited significantly increased percentage of Annexin V-positive cells (Fig. 1i,j), indicating increased apoptosis. Aβ42_O_ significantly enhanced MitoSox-Red and JC-1 intensity (Fig. 1a–h) and significantly increased the percentage of Annexin V-positive cells (Fig. 1i,j). However, PLD1 transfection or carbachol treatment, a PLD1 activator, significantly prevented the increase in Mitosox-Red and JC-1 intensity (Fig. 1a–h) as well as the percentage of Annexin V-positive cells induced by cofilin-S3A and Aβ42_O_ (Fig. 1i,j).

### PLD1 activity reciprocally promotes cofilin phosphorylation/inactivation

**Figure 2:**
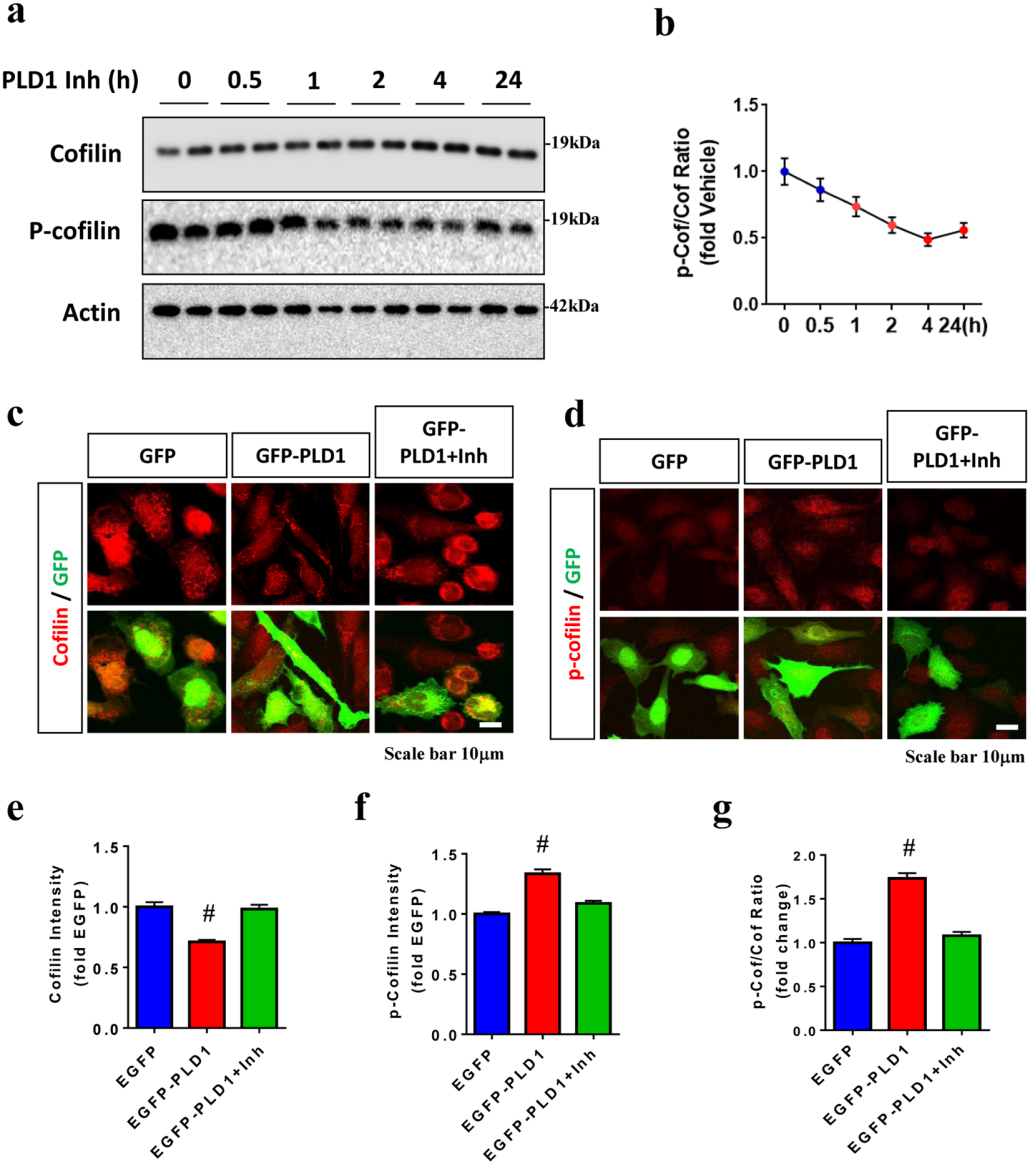
PLD1 activity reciprocally promotes cofilin phosphorylation. (**a**,**b**) HT22 cells treated with PLD1 inhibitor for the indicated times, and equal amount of cell lysates subjected to immunoblotting for cofilin, p-cofilin, and actin. Representative blots shown. (**b**) Quantitative graph showing the ratio of p-cofilin vs. cofilin with PLD1 inhibitor treatment over 24 h. (**c**–**g**). HT22 cells transfected with EGFP or EGFP-PLD1 for 48 h with treatment of PLD1 inhibitor VU0359595 (1 μM) or DMSO for 4 h and subjected to ICC for cofilin and p-cofilin followed by confocal imaging. (**c**,**d**) Representative images showing cofilin, p-cofilin, and EGFP fluorescence. (**e**–**g**) Quantitative graphs showing intensities of total cofilin, p-cofilin, and p-cofilin/cofilin ratio among EGFP+ cells normalized to EGFP control (*t*-test, ^#^P < 0.001, n = 6 replicates). All error bars represent S.E.M. Full-length blots are presented in Supplemental Fig. 7.

It has been reported that PLD1 activity requires PIP2^30, 31^, which is able to bind to dephosphorylated cofilin and inhibit its activity^32, 33^. Interestingly, PA, the product of PLD1, can stimulate the activity of PIP5K which synthesizes PIP2^34, 35^. Moreover, phosphorylated cofilin preferentially binds to PLD1 and stimulates its activity^25^. Therefore, we hypothesized that PLD1 may reciprocally regulate the activation status of cofilin either directly or indirectly. Thus, we treated HT22 cells with the potent PLD1 inhibitor VU0359595 (1 μM) for 0.5, 1, 2, 4, and 24 hours, and lysates were assessed for total cofilin, phospho-cofilin (p-cofilin), and actin. Surprisingly, PLD1 inhibitor gradually reduced the ratio of p-cofilin/Cofilin up to 4 hours (Fig. 2a,b). After 24 h, p-cofilin/cofilin ratio was still significantly reduced, albeit with a slight reversal (Fig. 2a,b). To determine whether PLD1 can promote cofilin phosphorylation in a different way, we employed the measurement of total and p-cofilin intensity in EGFP or EGFP-PLD1 transfected HT22 cells. PLD1-EGFP transfected cells exhibited a significant 25% decrease in total cofilin while showing a significant 25% increase in p-cofilin intensity (Fig. 2c–f), which resulted in a significant >70% increase in the ratio of p-cofilin/cofilin in PLD1-EGFP transfected versus EGFP cells (Fig. 2g). However, treatment of PLD1 inhibitor reversed both cofilin and p-cofilin back to control levels (Fig. 2c–g). Despite the reversal in the p-cofilin/cofilin ratio by PLD1 inhibition, the interaction between cofilin-S3E and PLD1 remained (Supplemental Fig. 2b), indicating that the PLD1/cofilin interaction is not sensitive to enzymatic PLD1 inhibition. These studies therefore indicate that, in addition to the role of p-cofilin in stimulating PLD1 activity, PLD1 activity reciprocally promotes cofilin phosphorylation/inactivation.

### Activated Cofilin preferentially forms a complex with p53 to promotes p53 localization to mitochondria and nucleus as well as apoptosis

**Figure 3:**
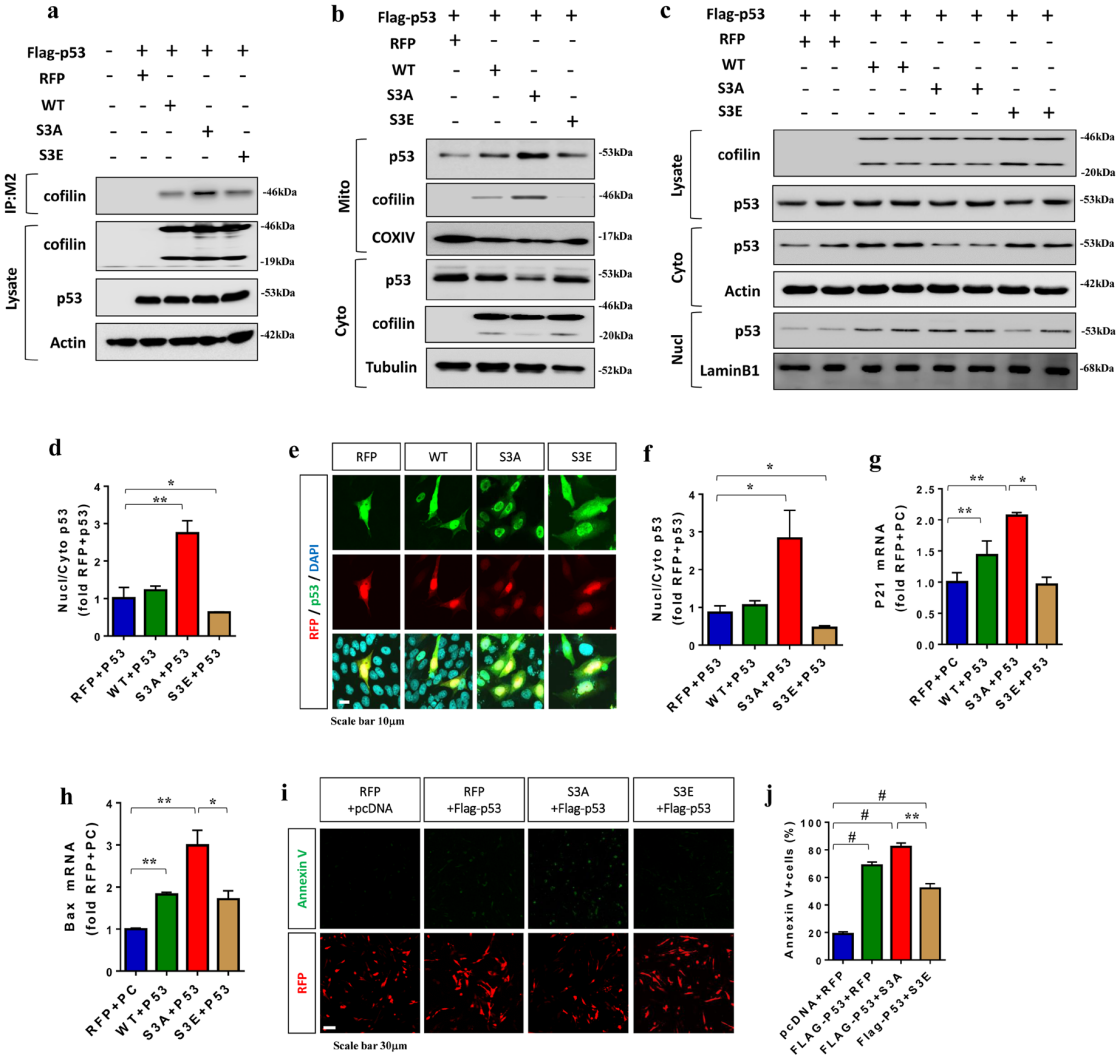
Activated Cofilin preferentially forms a complex with p53 to promote p53 localization to mitochondria and nucleus as well as apoptosis. (**a**) HEK293T cells co-transfected with Flag-p53 and mRFP, cofilin-mRFP (WT), cofilin-S3A-mRFP, or cofilin-S3E-mRFP for 36 h, and lysates subjected to immunoprecipation for Flag (M2) followed by immunoblotting for cofilin, p53, and actin. Blots from a representative experiment shown. (**b**) HT22 cells co-transfected with Flag-p53 and mRFP, cofilin-mRFP (WT), cofilin-S3A-mRFP, or cofilin-S3E-mRFP for 36 h and subjected to biochemical isolation of mitochondrial vs. cytosol fractions followed by immunoblotting for cofilin, p53 (Flag), COX IV, and tubulin. Blots from a representative experiment shown. (**c**,**d**) HT22 cells co-transfected with Flag-p53 and mRFP, cofilin-mRFP (WT), cofilin-S3A-mRFP, or cofilin-S3E-mRFP for 36 h and subjected to biochemical isolation of nuclear vs. cytoplasmic fractions followed by immunoblotting for cofilin, p53 (Flag), COX IV, actin, and LaminB1. Blots from a representative experiment shown. (**d**) Quantitative graph showing the ratio of p53 in nuclear vs. cytoplasmic fractions (1-way ANOVA, post hoc Tukey, ^*^P < 0.05, ^**^P < 0.005, n = 4 replicates). (**e**,**f**) HT22 cells co-transfected with Flag-p53 and mRFP, cofilin-mRFP (WT), cofilin-S3A-mRFP, or cofilin-S3E-mRFP and subjected to ICC for Flag-p53 (M2) followed by confocal imaging. Representative images showing mRFP, Flag-p53, and DAPI fluorescence. (f) Quantitative graph showing the ratio of Flag-p53 nuclear/cytoplasmic staining normalized to mRFP+ p53 control (1-way ANOVA, post hoc Tukey,^*^P < 0.05, ^**^P < 0.005, n = 6 replicates). (**g**,**h**) HT22 cells co-transfected with Flag-p53 and mRFP, cofilin-mRFP (WT), cofilin-S3A-mRFP, or cofilin-S3E-mRFP and subjected to total RNA isolation followed by qRT-PCR for Bax and p21 mRNA. Quantitative graphs showing p21 and Bax transcript levels normalized to mRFP+ PC (pcDNA vector) control (1-way ANOVA, post hoc Tukey, ^*^P < 0.05, ^**^P < 0.005, n = 6 replicates). (**i**,**j**) HT22 cells co-transfected with Flag-p53 and mRFP, cofilin-mRFP (WT), cofilin-S3A-mRFP, or cofilin-S3E-mRFP and subjected to Annexin-V staining followed confocal imaging. (**i**) Representative images showing Annexin V and mRFP fluorescence. **(j**) Quantitative graph showing the percentage of Annexin V+ cells among mRFP+ cells (1-way ANOVA, post hoc Tukey, ^*^P < 0.05, ^**^P < 0.005, n = 6 replicates). All error bars represent S.E.M. Full-length blots are presented in Supplemental Fig. 7.

Cofilin can enter into the nucleus by its active nuclear localization signal^36^, and activated/dephosphorylated cofilin under oxidative stress translocates to mitochondria, which induces mitochondrial dysfunction and apoptosis^37, 38^. As an actin-associated protein, cofilin has been shown to be an active carrier of actin into the nucleus^39^. Similarly, p53 can translocate into both nucleus and mitochondria to induce cell death and mitochondrial dysfunction^40, 41^. Interestingly, p53 nuclear translocation is associated with microtubule^21, 42^ and actin dynamics^43, 44^, the latter that is also associated with cofilin^45, 46^. Accordingly, we hypothesized that cofilin may form a complex with p53 and regulate its localization to mitochondria and/or nucleus. To determine whether cofilin forms a complex with p53, HEK293T cells were co-transfected with Flag-p53 and mRFP, cofilin-mRFP (WT), cofilin-S3A-mRFP, or cofilin-S3E-mRFP. In co-immunoprecipitation experiments, Flag-p53 immune complexes indeed contained cofilin, and cofilin-S3A was markedly increased in Flag-p53 immune complexes even though the levels of cofilin variants and p53 were virtually identical (Fig. 3a). Biochemical isolation of mitochondria versus cytosol from HT22 cells showed that cofilin-S3A reduced cytosolic p53 while increasing mitochondrial p53 (Fig. 3b). As expected, cofilin-S3A but not cofilin-S3E preferentially translocated to mitochondria (Fig. 3b).

To determine whether cofilin activation status also alters the nuclear localization of p53, we also subjected co-transfected HT22 cells to nuclear versus cytoplasmic isolation or whole cell lysis. Cofilin-S3A-mRFP transfection did not alter p53 levels in whole cell lysates but reduced cytoplasmic p53 and increased nuclear p53 compared to mRFP transfection, thereby significantly increasing the ratio of nuclear/cytoplasmic p53 (Fig. 3c,d). While the nuclear/cytoplasmic p53 ratio was unchanged by cofilin-mRFP (WT), cofilin-S3E-mRFP significantly reduced this ratio (Fig. 3c,d). To confirm these biochemical observations in a different way, we measured the intensity of nuclear versus cytoplasmic p53 by immunofluorescence in mRFP, cofilin-mRFP (WT), cofilin-S3A-mRFP, and cofilin-S3E-mRFP transfected cells. Quantification of immunofluorescence signals confirmed that S3A cofilin significantly increased while S3E decreased the ratio of nuclear/cytoplasmic p53 compared to mRFP control (Fig. 3e,f). To determine whether the changes in nuclear p53 result in the expected alterations in p53-induced nuclear transcription in HT22 cells, we performed real-time qRT-PCR for p21 and Bax, 2 p53-responsive genes known for their roles in apoptosis^47^. Co-transfection of p53 with cofilin-S3A significantly increased both p21 and Bax mRNA levels compared to co-transfection of p53 with cofilin-S3E or vector control (Fig. 3g,h), indicating that activated cofilin promotes p53-mediated nuclear transcription.

We next determined whether the changes in seen in HT22 cells are also observed in primary neurons. We transduced mouse cortical primary neurons on DIV2 with p53/GFP (non-fusion protein) lentivirus together with adenovirus expressing mRFP, cofilin-S3A-mRFP, or cofilin-S3E-mRFP. On DIV7, we subjected neurons to biochemical fractionation for mitochondria versus cytosol as well as nuclear versus cytoplasm. Indeed, we confirmed that cofilin-S3A but not cofilin-S3E increases both mitochondrial and nuclear p53 (Supplemental Fig. 3a–c). Even the absence of exogenous p53 expression, significant increases in endogenous mitochondrial and nuclear p53 were seen by the expression of cofilin-S3A but not cofilin-S3E (Supplemental Fig. 3d–f). Likewise, qRT-PCR experiments also confirmed that cofilin-S3A but not cofilin-S3E significantly increased p53-responsive transcripts Bax and p21 in primary neurons (Supplemental Fig. 3g).

Finally, to determine whether cofilin activation status modifies p53-dependent apoptosis, we subjected co-transfected HT22 cells to Annexin V staining. As expected, >60% of p53+mRFP transfected cells were already Annexin V-positive, and p53+cofilin-S3A-mRFP transfected cells displayed significantly higher percentage of Annexin V-positive cells (82%) (Fig. 3i,j). In contrast, p53+cofilin-S3E-mRFP transfected cells showed significantly reduced Annexin V-positive cells (~50%) compared to p53+mRFP transfected cells (Fig. 3i,j), indicating that expression of cofilin-S3E ameliorates p53-induced apoptosis.

### PLD1 activity negatively regulates cofilin-promoted p53 nuclear translocation

**Figure 4:**
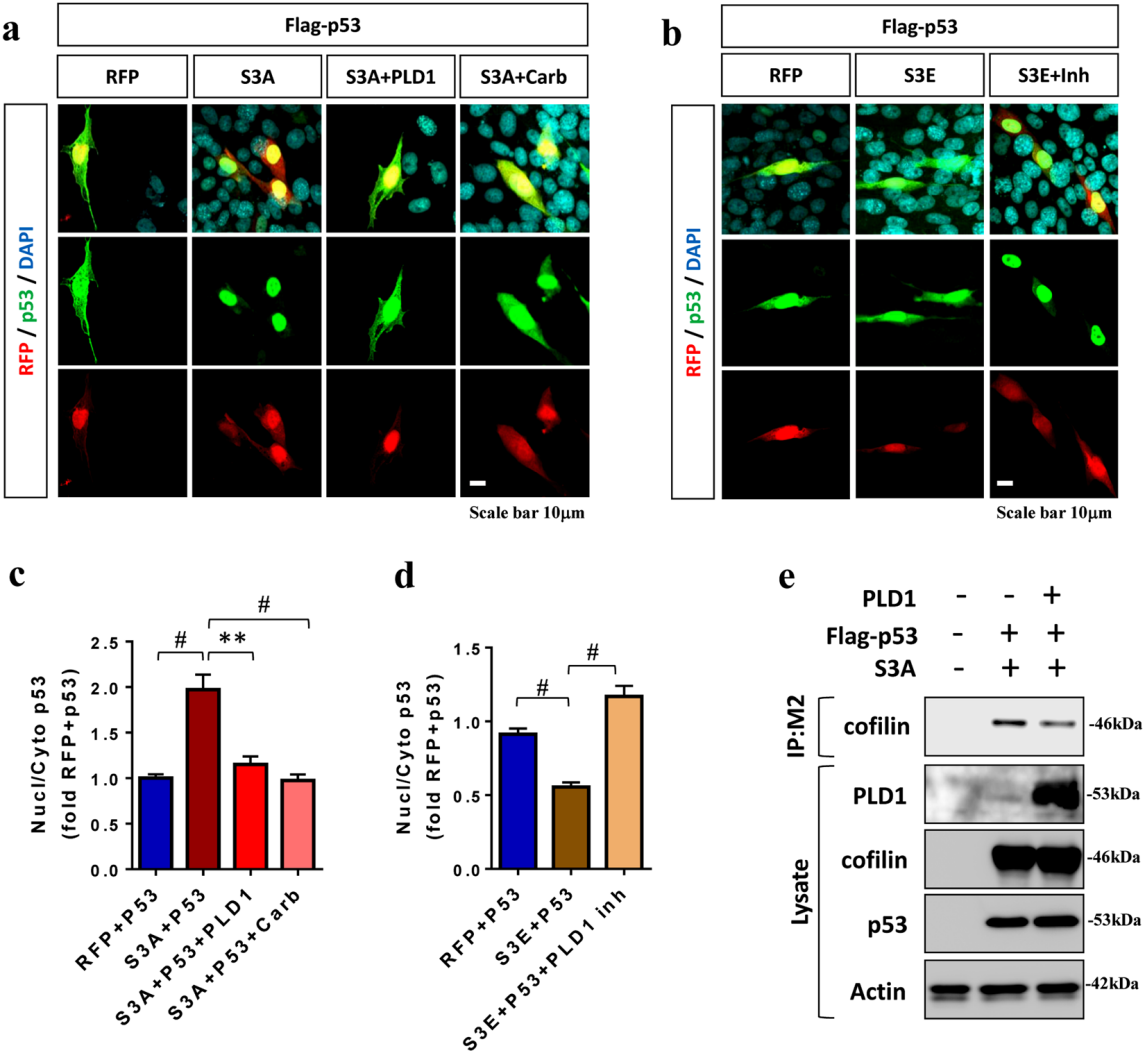
PLD1 activity negatively regulates cofilin-promoted p53 nuclear translocation. (**a**–**d**) HT22 cells co-transfected with Flag-p53 and mRFP, cofilin-mRFP (WT), cofilin-S3A-mRFP, or cofilin-S3E-mRFP for 4 h, treated with/without carbachol (1 mM) or PLD1 inhibitor (1 μM) for 24 h, and subjected to ICC for Flag-p53 (M2) followed by confocal imaging. (**a**,**b**) Representative images showing Flag-p53, mRFP, and DAPI fluorescence. (**c**,**d**) Quantitative graphs showing the ratio of nuclear/cytoplasmic p53 intensities normalized to mRFP+p53 control (1-way ANOVA, post hoc Tukey, ^**^P < 0.005, ^#^P < 0.0005, n = 6 replicates). (**e**) HEK293T cells co-transfected with/without Flag-p53, cofilin-S3A-mRFP, and/or PLD1 for 36 h, and lysates subjected to sonication and immunoprecipitation for Flag (M2) followed by immunoblotting for cofilin, p53 (M2), PLD1, and actin. A representative experiment shown. All error bars represent S.E.M. Full-length blots are presented in Supplemental Fig. 7.

We next determined whether PLD1 regulates cofilin-mediated control of p53 nuclear localization. As expected, cofilin-S3A-mRFP+p53 transfected HT22 cells showed significantly increased ratio of nuclear/cytoplasmic p53 compared to mRFP+p53 transfected cells (Fig. 4a,c). However, PLD1 transfection or carbachol treatment completely abrogated cofilin-S3A-dependent increase in the ratio of nuclear/cytoplasmic p53 (Fig. 4a,c). Conversely, cofilin-S3E-mRFP+p53 transfected cell showed significantly reduced ratio of nuclear/cytoplasmic p53 compared to mRFP+p53 transfected cells, but this reduction was completely reversed by PLD1 inhibitor treatment (Fig. 4b,d). In agreement with these results, PLD1 transfection reduced cofilin-S3A in p53 immune complexes in HEK293T cell, indicating that PLD1 interferes with p53/cofilin interaction (Fig. 4e). These results taken together underscore the role of PLD1 in negatively regulating the cofilin-p53 pathway.

### Endogenous cofilin promotes mitochondrial and nuclear localization of p53, p53-induced transcription, and p53-mediated cell death

**Figure 5:**
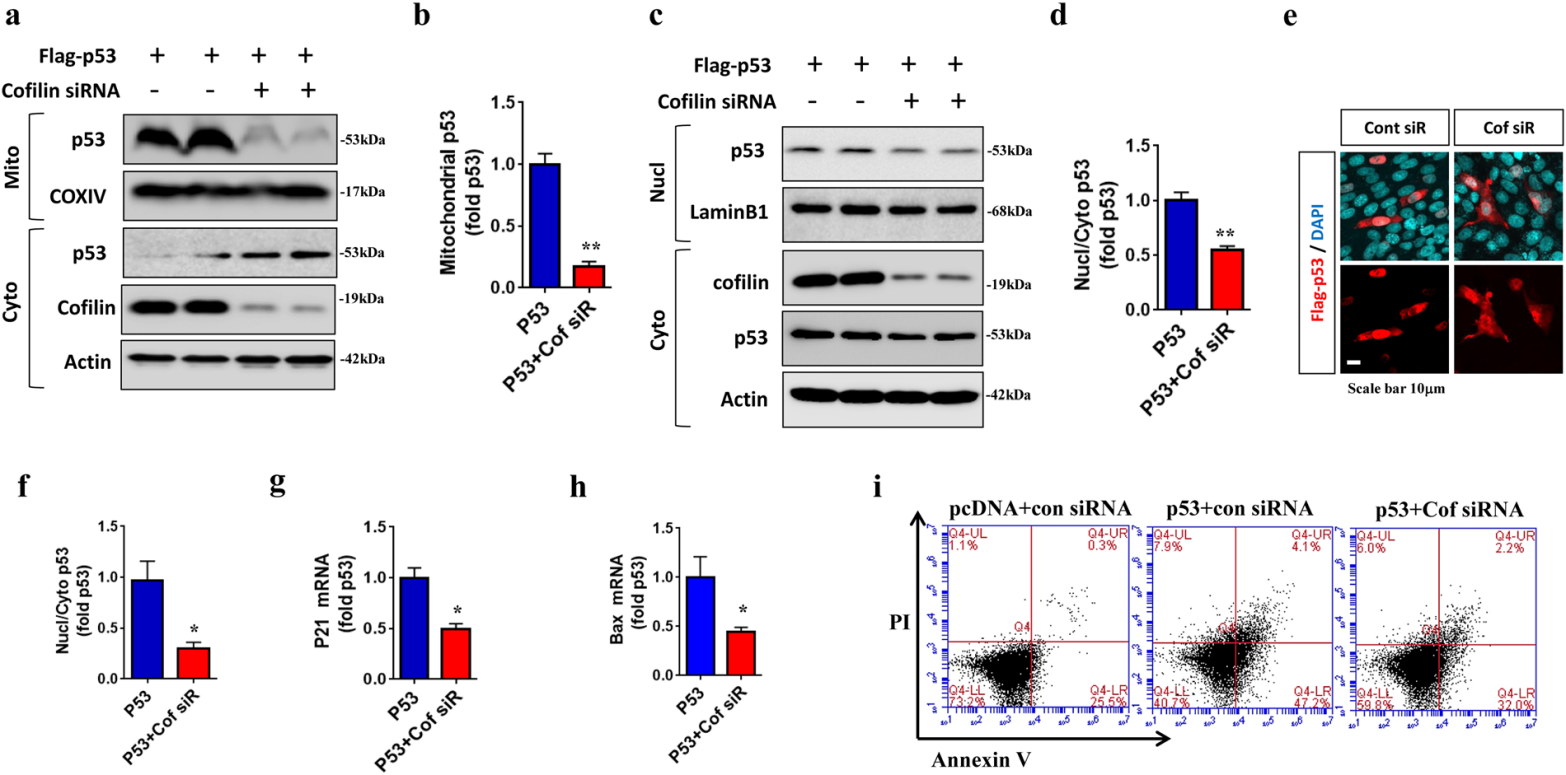
Endogenous cofilin promotes mitochondrial and nuclear localization of p53, p53-mediated nuclear transcription, and p53-induced cell death in HT22 cells. (**a**–**d**) HT22 cells co-transfected with Flag-p53 and control or cofilin siRNA for 48 h, and subjected to biochemical isolation of mitochondrial vs. cytoplasmic fractions or nuclear vs. cytoplasmic fractions followed by immunoblotting for p53, COXIV, cofilin, LaminB1, and actin. (**a**,**c**) Blots from representative experiments shown. (**b**,**d**) Quantitative graphs showing the level of mitochondrial Flag-p53 or the ratio of nuclear/cytoplasmic Flag-p53 normalized to p53 control (*t*-test, ^**^P < 0.005, n = 4 replicates). (**e**,**f**) HT22 cells co-transfected with Flag-p53 and control or cofilin siRNA for 48 h, and subjected to ICC for Flag-p53 (M2) followed by confocal imaging. (**e**) Representative images showing Flag-p53 and DAPI fluorescence. (**f**) Quantitative graph showing the ratio of nuclear vs. cytosol p53 intensity normalized to p53 control (*t*-test, ^*^P < 0.05, n = 6 replicates). (**g**,**h**) HT22 cells co-transfected with Flag-p53 and control or cofilin siRNA for 48 h, and subjected to total RNA isolation followed by qRT-PCR for Bax and p21 mRNA. Quantitative graphs showing p21 and Bax transcript levels normalized to p53 control. (**i**) HT22 cells co-transfected with Flag-p53 and control or cofilin siRNA for 48 h, and subjected to Annexin V and PI staining followed by FACS analysis. All error bars represent S.E.M. Full-length blots are presented in Supplemental Fig. 7.

While our data indicated that overexpression of activated cofilin amplifies the p53 pathway, it is possible that such activity could be an artifact of cofilin overexpression. As such, we performed cofilin knockdown experiments using siRNA transfection. siRNA knockdown of cofilin in HT22 cells dramatically reduced mitochondrial p53 by ~80% while increasing cytoplasmic p53 in biochemical fractionation experiments (Fig. 5a,b). Biochemical isolation of nuclear versus cytoplasmic fractions also demonstrated that cofilin siRNA knockdown significantly reduced the ratio of nuclear/cytoplasmic p53 (Fig. 5c,d). Similarly, cofilin siRNA transfection significantly reduced the immunofluorescence intensity ratio of nuclear/cytoplasmic Flag-p53 (Fig. 5e,f) and p53-responsive transcripts p21 and Bax (Fig. 5g,h). Likewise, genetic reduction of cofilin in *cofilin*+/− primary neurons showed a significant reduction in nuclear versus cytoplasmic p53 and p53-responsive genes Bax and p21 compared to littermate wild type neurons (Supplemental Fig. 4a–c). Finally, Annexin V/PI staining followed by FACS analysis in HT22 cells demonstrated that cofilin siRNA antagonizes p53-dependent increase in Annexin V+ (early apoptotic, lower right quadrant, 47.2% vs. 32%) and Annexin V+/PI+ (late apoptotic, upper right quadrant, 4.1% vs. 2.2%) cells (Fig. 5i). These results collectively show that endogenous cofilin normally promotes nuclear and mitochondrial localization of p53 and p53-dependent cell death.

### Genetic reduction in *cofilin* prevents the accumulation of mitochondrial and nuclear p53 in APP/PS1 transgenic mice

**Figure 6:**
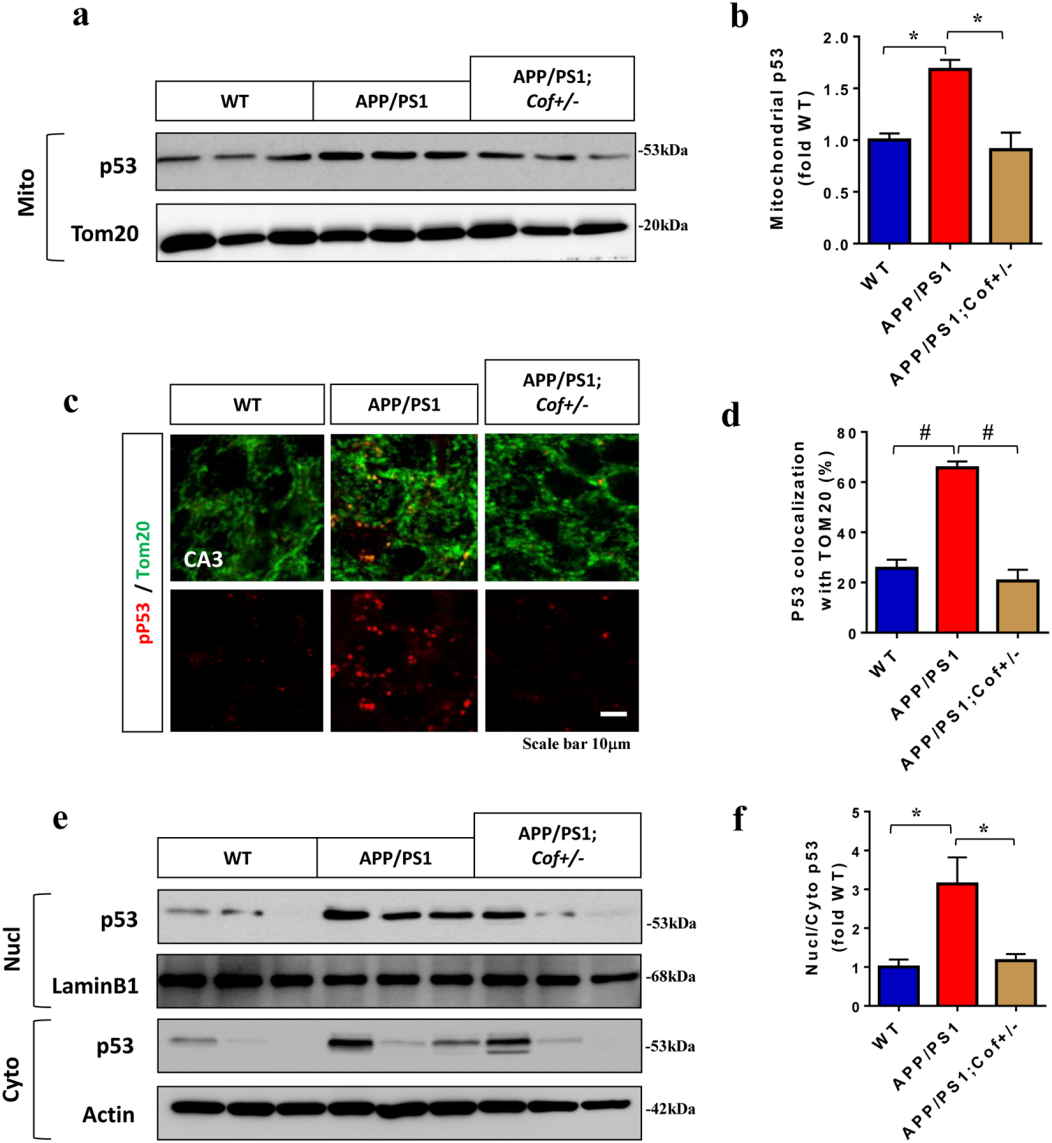
Genetic *cofilin* reduction prevents the increase in mitochondrial and nuclear localization of p53 in brains of APP/PS1 mice. (**a**,**b**) Dissected hippocampi from 5- month old WT, APP/PS1, and APP/PS1;*cofilin*+/− mice subjected to biochemical isolation of mitochondrial vs. cytoplasmic fractions and immunoblotting for p53, TOM20, and actin. (**a**) Representative blots shown. (**b**) Quantitative graph showing p53 in the mitochondrial fraction normalized to WT control (1-way ANOVA, post hoc Tukey, ^*^P < 0.05, n = 3 mice/grp). (**c**,**d**) Brain sections from 5-month old WT, APP/PS1 and APP/PS1;*cofilin*+/− littermate mice subjected to IHC for endogenous p53 and TOM20 followed by confocal imaging. (**c**) Representative images showing p53 and TOM20 fluorescence in CA3. (**d**) Quantitative graph showing the percentage colocalization of p53 with TOM20 performed by Image J normalized to WT control (1-way ANOVA, post hoc Tukey, ^#^P < 0.0005, n = 4 mice/grp). (**e**,**f**) Dissected hippocampi from 5- month old WT, APP/PS1, and APP/PS1;*cofilin*+/− mice subjected to biochemical isolation of nuclear vs. cytoplasmic fractions and immunoblotting for p53, LaminB1, and actin. (**e**) Representative blots shown. (**f**) Quantitative graph showing the ratio of endogenous p53 in nuclear vs. cytoplasmic fractions normalized to WT control (ANOVA, post hoc Tukey, ^*^P < 0.05, n = 3 mice/grp). All error bars represent S.E.M. Full-length blots are presented in Supplemental Fig. 7.

To confirm our findings in cultured cells, we utilized 5-month old wild type (WT), APP/PS1, and APP/PS1 mice lacking 1 copy of endogenous *cofilin* (APP/PS1;*cofilin*+/−). At 5 months of age, APP/PS1 mice exhibit low levels of Aβ deposition and are significantly compromised in hippocampal synaptic plasticity (i.e. LTP), the latter that is rescued by reduction in 1 copy of *cofilin* (APP/PS1;*cofilin*+/−)^1^. Biochemical isolation of mitochondria from dissected hippocampi showed that mitochondria of APP/PS1 mice contained significantly increased p53 compared to WT controls (Fig. 6a,b). However, mitochondrial p53 levels reverted back to WT levels in APP/PS1;*cofilin*+/− mice (Fig. 6a,b), indicating that normal level of endogenous cofilin is necessary to increase mitochondrial p53 in APP/PS1 mice. To confirm this finding in a different way, we subjected the aforementioned mice to immunohistochemistry (IHC) for p53 and Tom20, a mitochondrial marker. Overall, p53 levels were elevated in APP/PS1 hippocampus compared to both WT and APP/PS1;*cofilin*+/− hippocampi (Supplemental Fig. 5a). At higher magnifications, while very little p53 was detected in the nucleus of neurons in brain as previously observed^40, 48, 49^, p53 immunoreactivity appeared as irregularly shaped puncta which often colocalized with the mitochondrial marker Tom20 (Fig. 6c). These p53 puncta were present both in neurons and astrocytes as evidenced by their presence in both NeuN or GFAP-positive cells in the hippocampus (Supplemental Fig. 5b,c). Quantification of p53 colocalization with Tom20 in CA3 neurons by Image J demonstrated that while only ~20–23% of p53 colocalized with Tom20 in WT and APP/PS1;*cofilin*+/− mice, > 65% of p53 colocalized with Tom20 in APP/PS1 mice (Fig. 6c,d). Although IHC failed to clearly visualize p53 in the nucleus, we reasoned that biochemical isolation of nuclear versus cytoplasmic fractions would enable the detection of nuclear p53. We were able to detect p53 in both nuclear and cytoplasmic fractions, albeit to a variable extent despite the even loading controls (Lamin B1 and Actin) (Fig. 6e). However, the ratio of nuclear/cytoplasmic p53 was significantly elevated in the hippocampus of APP/PS1 mice compared to WT and APP/PS1;*cofilin*+/− mice (Fig. 6e,f), indicating that endogenous cofilin also promotes APP/PS1-induced nuclear p53 localization *in vivo*. Therefore, these studies collectively demonstrate that endogenous cofilin plays an integral role upstream of the p53 pathway, which significantly contributes to p53-mediated mitochondrial and nuclear cell death apparatus *in vitro* and *in vivo*.

### p53 is required for cofilin-induced cell death

**Figure 7:**
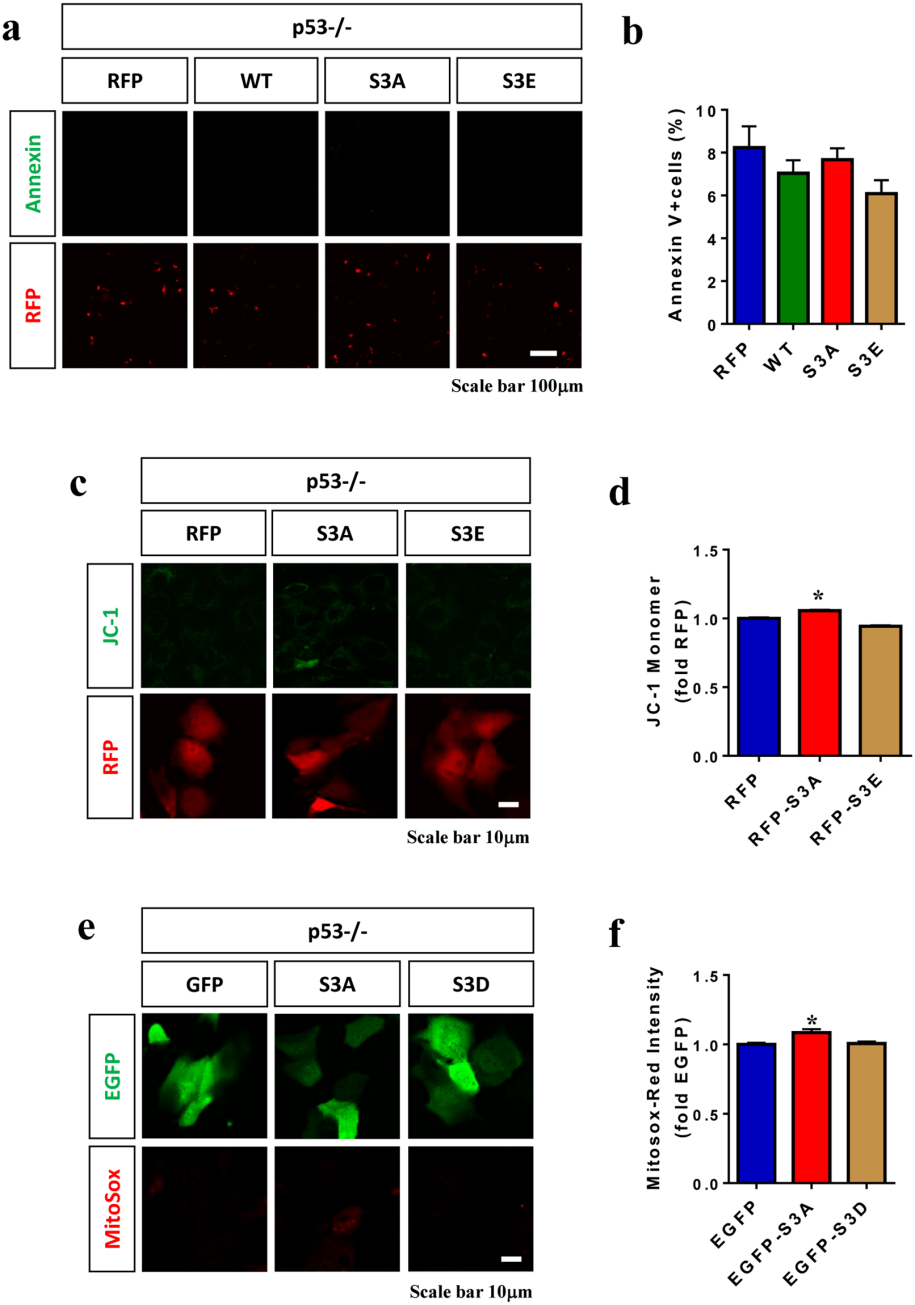
p53 is required for cofilin-induced cell death. (**a**–**d**) *p53*−/− MEF cells transfected with mRFP, cofilin-mRFP (WT), cofilin-S3A-mRFP, or cofilin-S3E-mRFP for 24 h, starved of FBS (1% FBS medium) for 24 h, and subjected to Annexin V or JC-1 staining followed by confocal imaging. (**a,c**) Representative images showing Annexin V, JC-1, and mRFP fluorescence. (**b**) Quantitative graph showing the percentage of Annexin V+ cells among mRFP+ cells (1-way ANOVA, not significant, n = 6 replicates). (**d**) Quantitative graph showing the intensity of JC-1 monomer staining (green) among mRFP+ cells (1-way ANOVA, post hoc Tukey, ^*^P < 0.05, n = 6 replicates). (**e,f**) *p53*−/− MEF cells transfected with EGFP, cofilin-S3A-EGFP, or cofilin-S3D-EGFP for 24 h, starved of FBS (1% FBS medium) for 24 h, and subjected to MitoSox-Red staining followed by confocal imaging. (**e**) Representative images of MitoSox-Red and EGFP fluorescence. (**f**) Quantitative graph showing the intensity of MitoSox-Red staining among EGFP+ cells (1-way ANOVA, post hoc Tukey, ^*^P < 0.05, n = 6 replicates). All error bars represent S.E.M.

Given the amplifying role of cofilin in p53-mediated cell death, we asked whether endogenous p53 is conversely required for cofilin-induced cell death. In *p53−/−* mouse embryonic fibroblasts (MEFs), transfection of WT, S3A, or S3E variants of cofilin-mRFP showed no significant changes in the percentage of Annexin V+ cells compared to mRFP control, even though transfected cells were readily detected by mRFP fluorescence (Fig. 7a,b). In all conditions, Annexin V+ cells remained low at 6.5–8% of transfected cells (Fig. 7b). In mitochondrial assays for JC-1 monomer and MitoSox-Red intensity, both JC-1 monomer and MitoSox-Red intensities were extremely low in all conditions (Fig. 7c,e). However, cofilin-S3A slightly but measurably increased JC-1 monomer and MitoSox-Red intensities compared to mRFP control (Fig. 7d,f), suggesting that cofilin may exert subtle mitochondrial toxicity independent of p53. In WT MEFs, in contrast, cofilin-S3A transfected cells exhibited significantly increased Annexin V+ cell death, mitochondrial superoxide (MitoSox-red) and JC-1 monomer compared to control or cofilin-S3E transfected cells (Supplemental Fig. 6a,b,c). Hence, these data indicate that while activated cofilin may produce subtle mitochondrial toxicity, endogenous p53 is required to execute the full apoptotic program even in the presence of activated cofilin.

## Discussion

**Figure 8:**
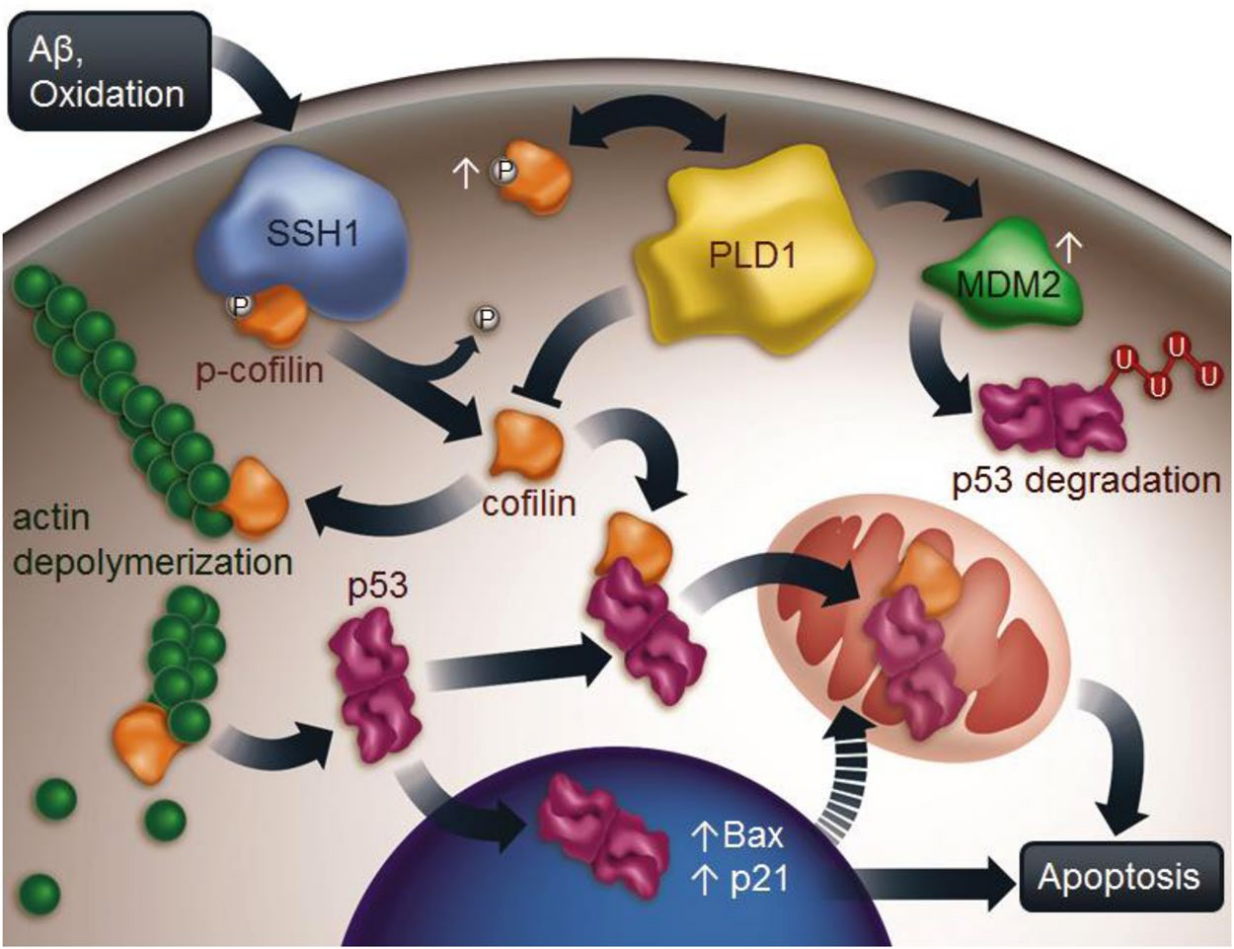
Schematic model of cofilin, p53, and PLD1 in the mediation, execution, and regulation of neuronal apoptosis in AD. Neurotoxic signals such as Aβ accumulation trigger oxidative stress and activate cofilin via its primary phosphatase SSH1^1^. Activated cofilin binds preferentially to p53 to promotes their localization to mitochondria (cofilin/p53 complex), which represents a direct path for mitochondria-mediated apoptosis. At the same time, cofilin enhances actin dynamics by severing/depolymerizing F-actin, leading to increased p53 translocation to the nucleus and transcription of p53-responsive targets genes (i.e. Bax & p21). As bax, p21, and others (i.e. PUMA & Foxo3a) are nuclear encoded but mitochondria-targeted pro-apoptotic proteins^23^, this represents a delayed nuclear path to reinforce mitochondria-mediated apoptosis. Although activated cofilin alone can induce subtle mitochondrial abnormalities, the cooption of the p53 mitochondrial and/or nuclear program is required to fully execute the apoptotic phenotype by activated cofilin. Meanwhile, phospho-cofilin can activate PLD1^25^, and PLD1 can also bind cofilin to inhibit cofilin/p53 complex formation and reciprocally inactivate cofilin (increase p-cofilin). Alternatively, PLD1 can enhance MDM2-dependent degradation of p53^26^, thereby representing a multi-brake mechanism for suppression of the cofilin-p53 apoptotic pathway.

Multiple studies have shown that activated cofilin can induce toxicity and cell death directly as a result of its translocation to mitochondria, leading to opening of the mitochondrial permeability transition pore (mPTP), reducing Δψm, releasing cytochrome c, and activating caspases^37, 50^. While these observations are consistent and reproducible across multiple studies, the critical involvement of p53 in this pathway has not been investigated. Moreover, while it has been reported that PLD1 preferentially associates with and is activated by p-cofilin, the impact of PLD1 activity on cofilin activation status and cofilin-induced cell death is unknown. In this study, we made a series of novel observations that fundamentally alter the mechanistic landscape underlying cofilin-mediated neuronal apoptosis. First, Aβ and/or S3A cofilin-induced toxicity (mitochondrial superoxide & reduction in Δψm) and cell death were rescued by enhancing PLD1 activity. PLD1 activity, in turn, reciprocally promoted cofilin phosphorylation, thereby essentially exerting a ‘double break’ on cofilin activation and downstream events. Second, S3A cofilin preferentially formed a complex with p53 and promoted the localization of p53 not only to mitochondria but also to the nucleus, resulting in further transcription of p53-responsive genes and promotion of p53-induced cell death. S3E or S3D cofilin, in contrast, failed to enhance mitochondrial translocation of p53 and significantly reduced nuclear localization of p53 and p53-induced cell death. Third, the effects of S3A and S3E in promoting and reducing the nuclear localization of p53 was abrogated by PLD1 activation and inhibition, respectively, which was associated with the ability of PLD1 to negatively regulate the cofilin/p53 complex. Fourth, knockdown of endogenous cofilin inhibited not only the mitochondrial but also nuclear translocation of p53, resulting in reduced p53 target gene transcription and cell death, indicating that cofilin normally functions as an important upstream mediator of the p53 cell death pathway. Indeed, we confirmed that endogenous cofilin is required for APP/PS1-induced elevation in p53 localization to mitochondria and nucleus *in vivo*, thereby highlighting the relevance of the cofilin-p53 pathway in AD pathogenesis. Finally, S3A cofilin, while able to slightly but measurably induce mitochondrial abnormalities (mito superoxide & in Δψm), was unable to induce cell death in cells genetically lacking *p53*, indicating that cofilin requires the cooption of the nuclear and/or mitochondrial p53 apoptotic program to fully execute this cell death pathway. These findings taken together indicate the critical roles of the cofilin-p53 pathway in the induction and execution of cell death, while PLD1 functions as a regulatory multi-brake system (Fig. 8).

In this study, we showed for the first time that cofilin forms a complex with p53, with S3A cofilin preferentially interacting with p53 and promoting the nuclear translocation of p53. This observation is consistent with previous studies showing that p53 nuclear translocation is associated with actin dynamics and that actin polymerization impairs the p53 nuclear import^19, 43^. Thus, in addition to its physical complex formation with p53, the ability of active cofilin to sever/depolymerize F-actin or compete with myosin II for regulating nuclear envelope tension^51^ contractility may play a crucial role in promoting p53 nuclear translocation (Fig. 8). Conversely, the ability of cofilin-S3E to dominantly interfere with F-actin depolymerization likely underlies its ability to partially block p53 nuclear translocation. Such nuclear/cytoplasmic localization of p53 was best associated with the degree of p53-responsive gene transcription (p21 & Bax) as well as apoptosis in both cofilin overexpression and knockdown paradigms. On the other hand, while cofilin-S3A promoted p53 mitochondrial translocation, cofilin-S3E did not interfere with this activity. This may be in part because oxidative mechanisms (i.e. cysteine oxidation of cofilin) are also associated with cofilin translocation to mitochondria, in addition to the distinct possibility that cofilin and p53 may not necessarily always translocate to mitochondria as a complex. The observation that knockdown of endogenous cofilin essentially abrogated p53 translocation to mitochondria coupled with observations with activated S3A cofilin, however, indicates that dephospho-cofilin is required for the efficient translocation of p53 to mitochondria.

It is well established that the p53 cell death pathway contains both a direct mitochondrial and an indirect nuclear component, the latter which also feeds back on mitochondria via nuclear activation of pro-apoptotic mitochondrial protein (i.e. Bax, p21, & PUMA)^52^. It has been shown that direct mitochondrial targeting of p53 can induce tumor suppressor activity and promote apoptosis in some tumors, whereas a nuclear component is necessary in neuronal cells^53, 54^. This study showed for the first time that S3A cofilin *per se* is insufficient to induce the full cell death program in the absence of *p53*. We hypothesize that the complete absence of S3A-induced cell death in *p53−/−* cells primarily results from the loss of nuclear p53 activity, since a small but significant mitochondrial perturbation by S3A was observed. However, it is entirely possible that a secondary p53-dependent mitochondrial component also contributes to the loss of S3A phenotype in *p53−/−* cells. Under stressful conditions, an initial mitochondrial component would be induced within minutes, whereas this insult would be reinforced via the nuclear component (i.e. Bax, p21, & PUMA) within hours, thereby culminating in the execution of the full cell death program. Thus, the loss of either the mitochondrial or nuclear component would be expected to antagonize cell death in a time- and stress-dependent manner. Likewise, our data indicate that the cofilin and p53 pathways are partially inseparable in both mitochondrial and nuclear pathways in that cofilin plays a key facilitator role in the induction phase, while p53 plays the decisive role to execute the full death program.

We and others have shown that upstream factors play crucial roles in the cofilin pathway. For example, RanBP9, a scaffolding protein that promotes Aβ production via scaffolding APP/BACE1 complexes^55^, also activates cofilin by enhancing SSH1^2^ while cooperating with a related tumor suppressor protein, p73, to promote nuclear and mitochondria-mediated apoptosis^50, 56^. We have also recently shown that Aβ-induced activation of cofilin requires SSH1^1^, one of the primary phosphatases to activate cofilin^57^. The observation that cofilin-S3A promoted mitochondrial and nuclear translocation of p53 and genetic reduction in *cofilin* abrogated the elevation in mitochondrial and nuclear p53 in APP/PS1 transgenic mice, underscore the importance of activated cofilin in the p53 apoptotic pathway (Fig. 8). Therefore, multiple upstream factors including RanBP9 and SSH1 are likely to converge on the cofilin-p53 pathway, whereas others (i.e. p73) may diverge away as either additive or reinforcing apoptotic routes. By contrast, PLD1 or carbachol antagonized Aβ- and/or cofilin-S3A-induced mitochondrial dysfunction and apoptosis while negatively regulating cofilin dephosphorylation (Fig. 8). Therefore, we attribute the protective role of PLD1, in part, to its reciprocal control of cofilin phosphorylation. In addition, it has been shown that PA, the product of PLD1, can be converted to PIP2^34, 35^, which inhibits the dephosphorylated form of cofilin^32, 33, 58^. Such actions may also explain our observation that PLD1 reduced the cofilin/p53 complex and inhibited cofilin-S3A-induced nuclear translocation of p53. A mutually nonexclusive explanation for the protective role of PLD1 may also be via MDM2, as PLD1 can elevate the level of MDM2, an E3 ligase that targets p53 for degradation^26^ (Fig. 8). Thus, the protective actions of PLD1 may come at multiple levels, including the previous observation that PLD1 reduces Aβ production by destabilizing the γ-secretase complex^50^. As such, strategies of enhancing PLD1 activity and/or inhibiting cofilin activation may hold therapeutic potential against AD and neurodegeneration.

## Materials and Methods

### Ethics approval

All the experiments methods and protocols involving mice used in this study were approved by the Institutional Animal Care and Use Committee (IACUC) at the University of South Florida (USF). All methods were performed in accordance with the relevant guidelines and regulations approved by IACUC and Institutional Biosafety Committees (IBC) at the USF.

### Mice

WT, APP/PS1 and APP/PS1;*cofilin*+/− mice were bred in the C57Bl6 background as previously described^1^. Mice were housed together until the time they were sacrificed at 5 months of age. Water and food were supplied *ad libitum* with 12-hour light/dark cycle under standard vivarium conditions.

### Cell culture

Mouse hippocampus-derived HT22 neuroblastoma cells^1^, human embryonic kidney cell line HEK293T, and mouse embryonic fibroblast *p53−/−* cells^59^ were cultured in Dulbecco’s modified Eagle’s medium (DMEM, Thermo Scientific, MA, USA) supplemented with 10% fetal bovine serum (FBS) and 1% penicillin/streptomycin (P/S). Mouse hippocampal primary neurons from P0 pups were cultured in Neurobasal medium supplemented with 1 × B-27 supplement and 1 × L-Glutamine (Invitrogen, CA, USA). Cells were cultured in a humidified atmosphere (5% CO_2_) at 37 °C^2^.

### Antibodies and reagents

Mouse anti-M2, anti-NeuN, anti-GFAP and β-actin monoclonal antibody was obtained from Sigma-Aldrich (St. Louis, MO, USA). Anti-cofilin (D3F9), p-cofilin, COXIV, Tom20, laminB1 and p53 (1C12) primary antibodies were purchased from Cell Signaling (Danvers, MA, USA). Anti-PLD1 and tubulin antibodies were purchased from Santa Cruz (Dallas, TX, USA). Carbachol and PLD1 inhibitor VU0359595 were purchased from Sigma-Aldrich (St. Louis, MO, USA). Aβ1-42 peptide was purchased from American Peptide (Sunnyvale, CA, USA). To prepare oligomeric A*β*1-42, the powder of A*β*1-42 was dissolved in HFIP at 1 mM. And then the A*β*1-42 in HFIP was evaporated in fume hood for overnight and subjected to speed vacuum for 1 h. Then A*β*1-42 film was dissolved in DMSO (5 mM), and PBS was added to a final concentration of 100 *μ*M A*β*1-42 and incubated at 4 °C for 24 h allowing it to form oligomers of A*β*1-42^1^.

### DNA construct and siRNA

pcDNA-Flag-p53 construct was obtained from Dr. Jiandong Chen^59^ (H. Lee Moffitt Cancer Center, Tampa, FL). PCGN-PLD1 and EGFP-PLD1 constructs were obtained from Dr. Michael A. Frohman^60^ (Stony Brook University, NY). mRFP, cofilin-mRFP, cofilin-S3A-mRFP and and cofilin-S3E-mRFP constructs were obtained from Dr. James Bamburg^3^ (Colorado State University, CO). The siRNA duplexes (19 nt) targeting cofilin (5′-GGAGGACCUGGUGUUCAUC-3′), and siRNA duplexes (19nt) targeting p53 (5′-CCACUUGAUGGAGAGUAUU-3′) were obtained from GE Dhamacon (Lafayette, CO, USA).

Generation of TP53-GFP lentivirus and cofilin adenovirus. Human TP53-GFP lentivirus plasmid was purchased from Abm (# LV342079, Richmond, BC, Canada). Lentivirus vectors were co-transfected with pVSVG and pAX2 using polyethylenimine (PEI) in HEK293T cells overnight. Media was replaced with serum free media. After 3 days, the conditioned media was collected and centrifuged to remove cell debris. The syringe filter (0.2–0.45 μM) was used to filter the viral medium. The virus in the medium was aliquoted, and frozen in −80 °C. Adenoviruses expressing mRFP, cofilin-S3A-mRFP and and cofilin-S3E-mRFP were obtained from Dr. James Bamburg (Colorado State University, CO).

### Transient transfections

Transient transfections of HT22, HEK293T or *p53*−/− MEF cells with DNA plasmids were performed using fugene HD (Promega, Madison, WI, USA) and Opti-MEM I (Invitrogen, Carlsbad, CA, USA) according to the manufacturer′s instructions. For siRNA transfections, lipofectamine 3000 (Invitrogen, CA, USA) and Opti-MEM I were applied and siRNA was transfected twice every 24 h. After four to six hours transfections, the medium was replaced with new complete medium. Generally, cells were incubated 24–48 h for plasmid transfections and 72 h for siRNA transfections prior to harvest.

### Immunocytochemistry and Immunohistochemistry

For immunocytochemistry (ICC), cells were washed with PBS and fixed at room temperature for 15 min with 4% paraformaldehyde (PFA). After washing with PBS, fixed cells were incubated with blocking solution containing 0.3% Triton X-100, 3% bovine serum albumin for 1 h, followed by overnight incubation at 4 °C with indicated primary antibodies. After three washes with PBST, cells were incubated for 2 h with Alexa-488 or Alexa-594 conjugated secondary IgG antibodies (Vector Laboratories, Burlingame, CA). Slides were then washed three times with PBST and mounted with fluorochrome mounting solution (Vector Laboratories). For immunohistochemistry (IHC), mice were perfused with PBS, and half brains were immediately stored at −80 °C for biochemical analysis, and the other half was fixed with 4% paraformaldehyde at 4 °C for 24 h followed by cryoprotection in 30% sucrose. Thirty-micron sections were blocked using normal goat serum for 1 h and subjected to primary antibodies (anti-P53 1:100 Cell Signaling, Danvers, MA, USA; anti-Tom20 1:50, Santa Cruz, USA) at 4 °C for overnight, followed by secondary antibody (Alexa-594 and Alexa-488) incubation for 1 h at room temperature prior to mounting. Images were captured with the Olympus FV10i confocal microscope (Tokyo, Japan), and the immunoreactivities were quantified from hippocampus region using the Image J software (National Institutes of Health, Bethesda, MD). Immunoreactivities were quantitated from every 12th serial section through an entire hippocampus. In ICC and IHC experiments, all comparison images were acquired with identical laser intensity, exposure time, and filter. Adjustments to the brightness/contrast were applied equally to all comparison images. Regions of interest were chosen randomly, and investigators were blinded to genotypes of mice and experimental conditions during image acquisition and quantification.

### Apoptosis and mitochondrial health assays

HT22 cells or *p53*−/− MEF cells were cultured in 24-well plates with glass cover slips or glass bottom 35mm dishes (both coated with fibronectin). Transfections were performed using fugene (Promega, Madison, WI) according to the manufacturer’s instruction, and (or) cells were incubated in the medium with 1%FBS for expecting time. For Annexin V (BD Biosciences, San Jose, CA), JC-1 (Invitrogen, Carlsbad, CA), and MitoSox-Red (Invitrogen, Carlsbad, CA) staining, live cell were washed with PBS and stained with Annexin V-FITC, JC-1, MitoSox-Red for 20 minutes. Cells were washed one time with binding buffer, and images were captured by Olympus FV10i confocal microscope (Tokyo, Japan). For Annexin V/PI cell death assays using FACS, the Annexin-V-FITC and PI Apoptosis Detection Kit (BD Bisosciences, San Jose, CA) was used followed by Accuri C6 flow cytometer analysis (BD biosciences, San Jose, CA) according to the manufacturer’s instructions.

### Immunoblotting

Cell lysate was lysed with RIPA lysis buffer (50 mM Tris pH 7.4, 150 mM NaCl, 2 mM ethlenediaminetetraacetic acid, 1% NP-40, 0.1% sodium dodecyl sulfate). Nuclear isolation and mitochondrial isolation were performed using the nuclear isolation kit and mitochondrial isolation kit for cell and for tissue (Thermo Scientific, Rockford, IL, USA), respectively, according to the manufacturer’s instructions. Total protein concentrations were quantified by a colorimetric detection assay (BCA Protein Assay, Pierce, USA). Equal amounts of protein lysates were separated by sodium dodecyl sulfate-polyacrylamide gel electrophoresis, and transferred to Immobilon-P membranes (Millipore Corporation, Bedford, MA, USA). Interested proteins were probed by primary antibodies and corresponding peroxidase-conjugated secondary antibodies, followed by detection by ECL (Merck Millipore Corporation, Darmstadt, Germany) and capture using the LAS-4000 imager (GE Healthcare Biosciences, Pittsburgh, PA).

### Quantitative real time RT–PCR

Quantitative real-time PCR was performed using Roche LightCycler® 96 System (Life Science, San Francisco, CA, USA), following the manufacturer’s recommended conditions. Total RNA was isolated from transiently transfected cells using Trizol reagent (Invitrogen, CA, USA), reverse transcribed (Superscript III, Invitrogen, CA,USA), and subjected to quantitative PCR analysis using Syber green master mix (Invitrogen, CA, USA). The comparative threshold cycle (Ct) value was used to calculate the amplification factor, and the relative amount of targets was normalized to GAPDH levels as we previously described^50^. The primer sequences are as follows: Bax—forward 5′-CCGGCGAATTGGAGATGAACT-3′and reverse: 5′-CCAGCCCATGATGGTTCTGAT-3′. GAPDH-forward 5′-TGTGTCCGTCGTGCATCTGA-3′ and reverse 5′-CCTGCTTCACCACCTTCTTGA-3′. p21—forward 5′-GAACTTTGACTTCGTCACGGAGA-3′ and reverse 5′-CTCCGTTTTCGGCCCTGAGA-3′. Primers were ordered from Integrated DNA Technologies (Coralville, IA, USA).

### Statistical analysis

All graphs were analyzed and made using GraphPad Prism 6.0 software (GraphPad Software, San Diego, CA, USA) using student′s *t*-test or one-way analysis of variance (ANOVA) followed by Tukey post hoc test. Differences were deemed significant when *P* < 0.05. All graphs were expressed as mean ± S.E.M (error bars).

### Availability of data and materials

All data generated or analyzed in this study are included in this article and supplementary files.

## Electronic supplementary material

Supplementary Information
