# Supplementary material for: Cofilin-mediated Neuronal Apoptosis via p53 Translocation and PLD1 Regulation
Source: Sci Rep. 2017 Sep 14;7:11532. doi: 10.1038/s41598-017-09996-3 (PMC5599510; doi:10.1038/s41598-017-09996-3)

Cofilin-mediated Neuronal Apoptosis via p53 Translocation and PLD1 Regulation

Tian Liu[1], Fang Wang[1], Patrick LePochat[1], Jung-A A. Woo[1],

Mohammed Zaheen Bukhari[1], Kyung Woo Hong[1],

Courtney Trotter[1], and David E. Kang[1,2,*]

[1] USF Health Byrd Alzheimer's Institute, Department of Molecular of Medicine, University of South Florida, Morsani College of Medicine, Tampa, FL 33613, USA. [2]James A. Haley Veteran's Administration Hospital, Tampa, FL 33612, USA. [*]Correspondence and request for materials should be addressed to D.E.K. (email: dkang@health.usf.edu)

Supplementary Information

Active Cofilin (S3A) promotes and inactive Cofilin (S3E) protects against DNA condensation and Aβ-induced cell death. Previous studies have shown that activated cofilin promotes mitochondria-mediated cell death, whereas inactive cofilin protects against cells death[1, 2]. To confirm whether cofilin activation status differentially alters toxicity in the mouse hippocampus-derived HT22 cell line, we transfected HT22 cells with mRFP, mRFP-cofilin wild type (WT), mRFP-cofilin S3A (constitutively active) or mRFP-cofilin S3E (constitutively inactive) for 24 hours, followed by treatment with or without 1 μM Aβ1-42 oligomers (Aβ42_O) for 24 hours. Cells transfected with cofilin-S3A but not cofilin-S3E showed greatly increased nuclear condensation (to ~45% of cells) compared to mRFP transfected cells (~5% of transfected cells) even without Aβ42_O treatment. As expected, Aβ42_O treatment significantly increased DNA condensation in RFP transfected cells and further increased DNA condensation in cofilin-S3A transfected cells (Supplemental Fig. 1a,b). Notably, however, cofilin-S3E transfected cells did not show significantly increased DNA condensation (~8% of transfected cells) compared to mRFP transfected cells, and Aβ42_O treatment did not significantly increase DNA condensation in cofilin-S3E transfected cells (~10% of transfected cells), indicating that the dominant negative effect of the constitutively inactive cofilin protects against Aβ42_O-induced toxicity (Supplemental Fig. 1a,b).

Supplemental Methods

Nuclear DNA condensation assay. HT22 cells cells were cultured in 24-well plates with glass cover slips or glass bottom 35mm dishes (both coated with fibronectin). Transfections were performed using fugene (Promega, Madison, WI) according to the manufacturer's instruction, and (or) cells were incubated in the medium with 1% FBS for expecting time. For DNA condensation assay, cells were washed with PBS and fixed at room temperature for 15 min with 4% paraformaldehyde (PFA). After washing with PBS, fixed cells were incubated with Hoechst33342 diluted in PBS (1:1000) for 20 min

followed by three washes using PBS. Images were then captured by Olympus FV10i confocal microscope (Tokyo, Japan). Cells with nuclear DNA condensation were counted from transfected cells, and the ratio of the number of cells with DNA condensation per transfected cells was used as the percentage of nuclear DNA condensation.

Supplemental References

1. Woo JA, Zhao X, Khan H, Penn C, Wang X, Joly-Amado A*, et al.* Slingshot-Cofilin activation mediates mitochondrial and synaptic dysfunction via Abeta ligation to beta1-integrin conformers. *Cell death and differentiation* 2015, **22**(6)**:** 921-934.

2. Chua BT, Volbracht C, Tan KO, Li R, Yu VC, Li P. Mitochondrial translocation of cofilin is an early step in apoptosis induction. *Nat Cell Biol* 2003, **5**(12)**:** 1083-1089.

Supplemental Legends

Supplemental Figure 1. **Active Cofilin (S3A) promotes and inactive Cofilin (S3E) protects against DNA condensation and Aβ-induced cell death**. (**a,b**) Hippocampus-derived HT22 neuroblastoma cells transfected with mRFP, cofilin-mRFP, cofilin-S3A-mRFP, or cofilin-S3E-mRFP for 24h and treated with/without 1μM Aβ1-42 oligomers for 24h. (**a**) Representative images of cells DAPI and RFP fluorescence showing DNA condensation (red arrows) by confocal microscope. (**b**) Quantitative graph showing percentage of RFP+ cells with DNA condensation (1-way ANOVA, post hoc Tukey, $^{\#}P<0.0005$, n=6 replicates). All error bars represent S.E.M.

Supplemental Figure 2. PLD1 inhibitor does not alter the interaction between PLD1 and phospho-cofilin (S3E). (**a**) Hippocampus-derived HT22 neuroblastoma cells transfected with EGFP and EGFP-PLD1 for 48h. Equal amount of cell lysates were subjected in immunoblotting for PLD1 and β-actin. (**b**) HEK293T cells co-transfected with/without EGFP-PLD1 and cofilin-S3E-mRFP for 36h followed by the treatment of PLD1 inhibitor VU0359595 (1 μM) or DMSO for 12h, and lysates subjected to immunoprecipation for mRFP or cofilin followed by immunoblotting for cofilin, PLD1, and actin. Blots from a representative experiment shown.

Supplemental Figure 3. Activated cofilin promotes mitochondrial and nuclear translocation of p53 and augments the transcription of p53-responsive genes in primary neurons. (**a-c**) Wild type primary cortical neurons were transduced with adenoviruses expressing mRFP, S3A-mRFP or S3E-mRFP together with lentivirus expressing p53-GFP (non-fusion protein) on DIV2. On DIV7, neurons were subjected in mitochondrial vs. cytoplasm isolation as well as nuclear vs. cytoplasm isolation. Equal amount of proteins from each fraction were subjected in immunoblotting for p53, lamin B1, β-actin Tom20, and cofilin variants. (**b,c**) Quantification of nuclear/cytoplasmic p53 and mitochondrial p53 normalized to control (1-way ANOVA, post hoc Tukey, *P<0.05, **P<0.005, n=4 replicates). (**d-f**) Wild type primary cortical neurons were transduced with adenoviruses expressing mRFP, S3A-mRFP or S3E-mRFP on DIV2 without exogenous p53. On DIV7, neurons were subjected in mitochondrial vs. cytoplasm isolation as well as nuclear vs. cytoplasm isolation. Equal amount of proteins from each fraction were subjected in immunoblotting for endogenous p53, lamin B1, β-actin, Tom20, and cofilin variants. (**e,f**) Quantification of endogenous nuclear/cytoplasmic p53 and endogenous mitochondrial p53 normalized to RFP control (1-way ANOVA, post hoc Tukey, *P<0.05, **P<0.005, n=4 replicates). (**g**) Wild type primary cortical neurons were transduced with adenoviruses expressing mRFP, S3A-mRFP or S3E-mRFP together with lentivirus expressing p53-GFP (non-fusion protein) on DIV2. On DIV7, neurons were subjected in qRT-PCR for Bax and p21 transcript and normalized to RFP+p53 control (1-way ANOVA, posthoc Tukey, *P<0.05, n=6 replicates). All error bars represent S.E.M.

Supplemental Figure 4. Genetic reduction in *cofilin* attenuates the nuclear translocation of p53 and transcription of p53-responsive genes in primary neurons. (a-c) Primary cortical neurons (WT and *Cofilin+/-*) were transduced with lentivirus expressing p53-GFP (non-fusion protein) on DIV2. (**a**) On DIV7, neurons were subjected in the nuclear vs. cytoplasm isolation. Equal amount of proteins from each fraction were subjected in immunoblotting for p53, lamin B1, β-actin, and cofilin. Representative blots shown. (**b**) Quantification of nuclear/cytoplasmic p53 ratio normalized to control (*t*-test, **P<0.005, n=6

replicates). **(c)** On DIV7, neurons were subjected in qRT-PCR for Bax and p21 transcripts, and quantifications are normalized to WT control neurons (*t*-test, *P<0.05, n=6 replicates). All error bars represent S.E.M.

Supplemental Figure 5. Immunohistochemical distribution of p53 in brains of WT, APP/PS1, and APP/PS1;*cofilin+/-* mice. Brain tissues from 5-month old WT, APP/PS1, and APP/PS1;*cofilin+/-* littermate mice were subjected in immunohistochemistry for (**a**) p53, tom20 and DAPI; (**b**) p53 and NeuN (CA3 region); (**c**) p53 and GFAP (CA3 region).

Supplemental Figure 6. Activated cofilin (S3A) induces cell death and mitochondrial dysfunction in WT MEF cells. (a-f) WT MEF cells were transfected with RFP (or EGFP), S3A-RFP (or S3A-EGFP) or S3E-RFP (or S3D-EGFP) for 48h, and cells were subjected to Annexin V, JC-1, or mitosox-red staining. (**a-c**) Representative images shown. (**d-f**) Quantification of Annexin V+ cells, JC-1 monomer intensity, and mitosox-red instensity among transfected cells (RFP+ or GFP+ cells) and normalized to controls (1-way ANOVA, post hoc Tukey, **P<0.005, n=6 replicates). All error bars represent S.E.M.

Supplemental Fig. 1

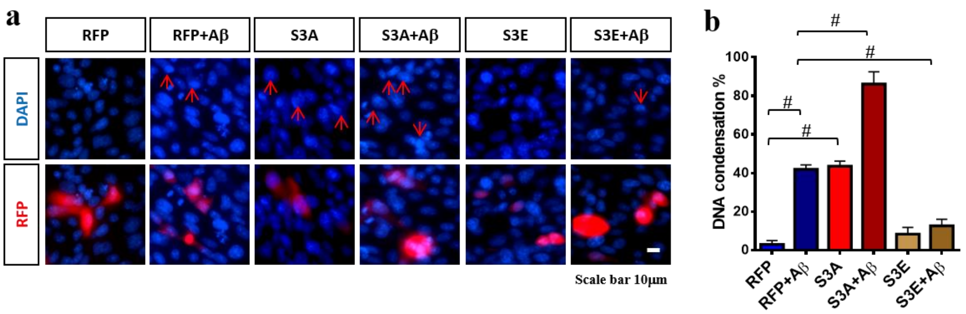

Supplemental Fig. 2

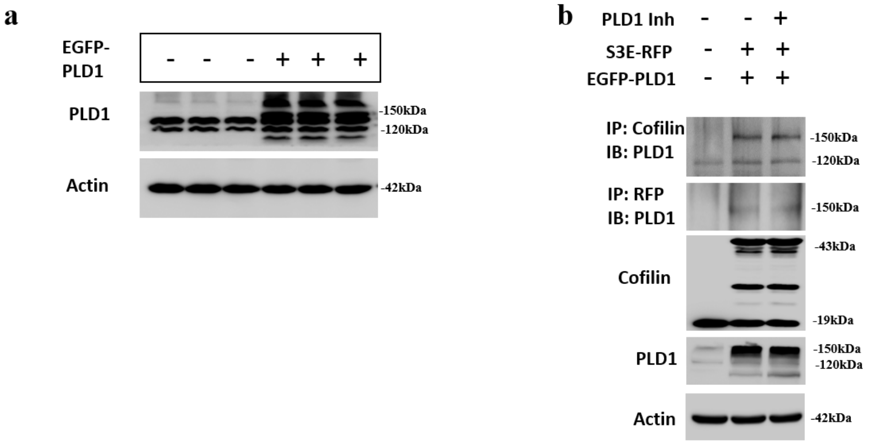

Supplemental Fig. 3

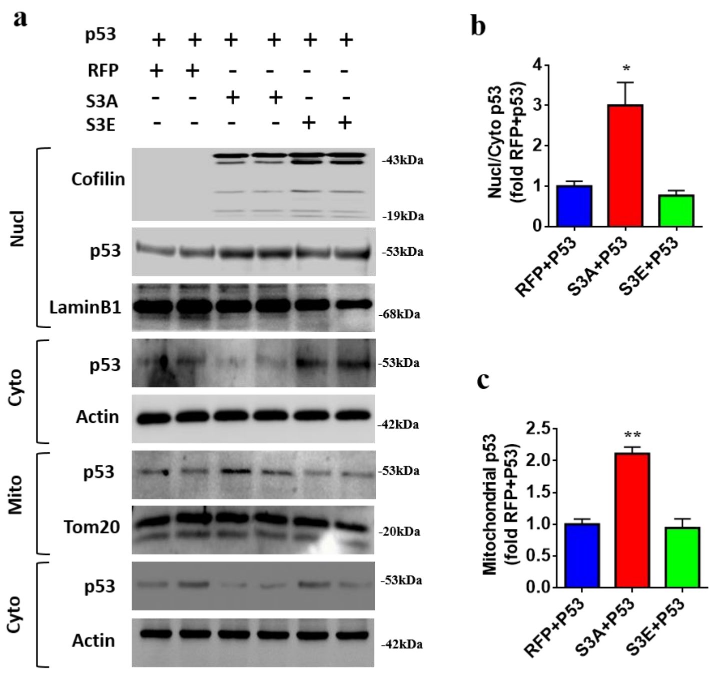

Supplemental Fig. 3 continued

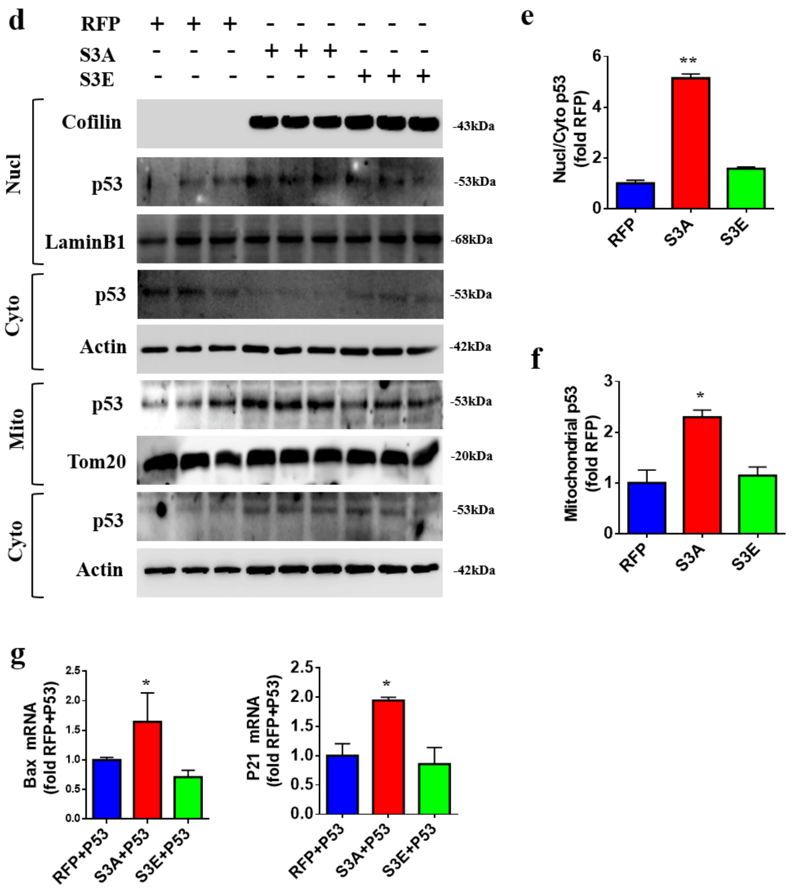

Supplemental Fig. 4

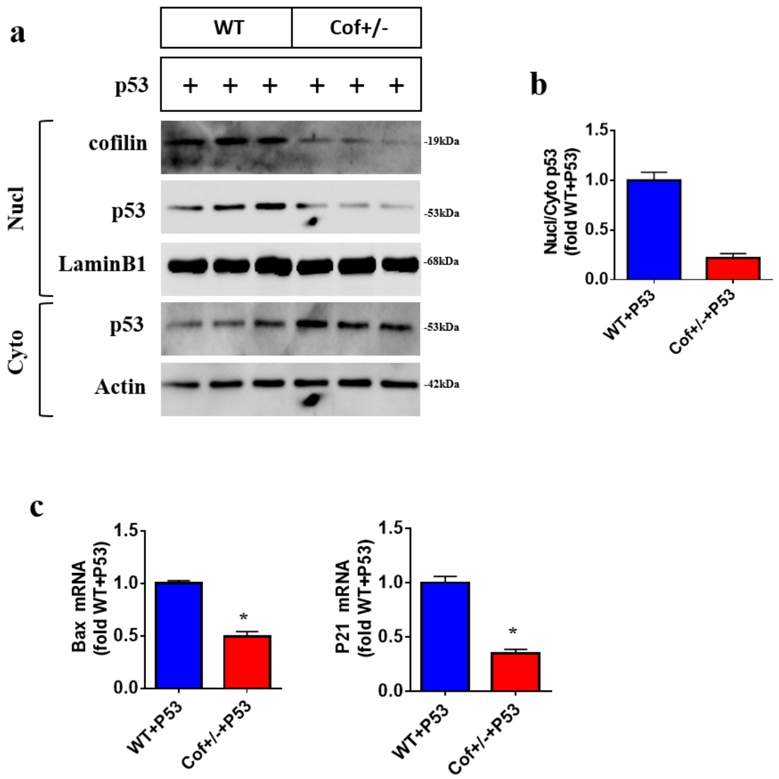

Supplemental Fig. 5

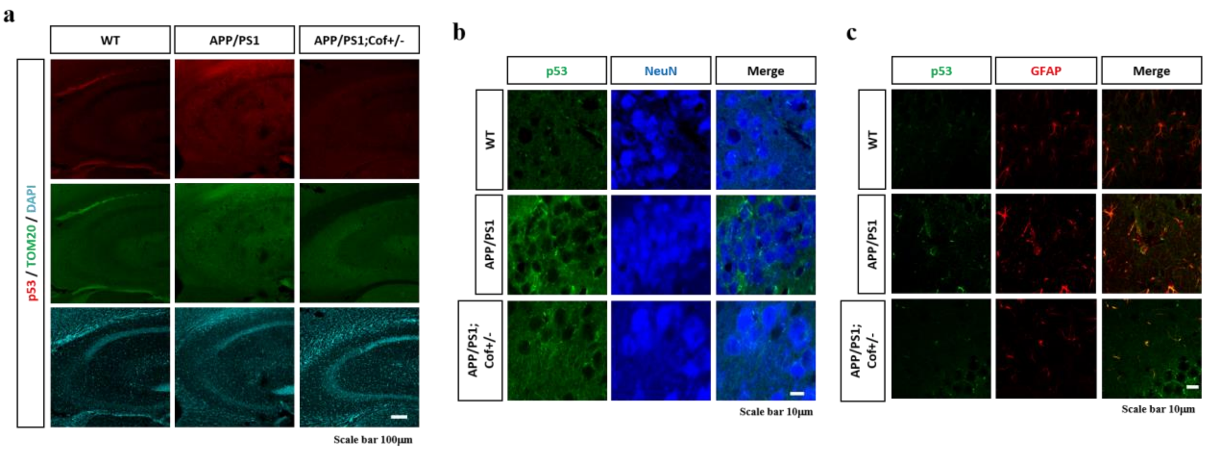

Supplemental Fig. 6

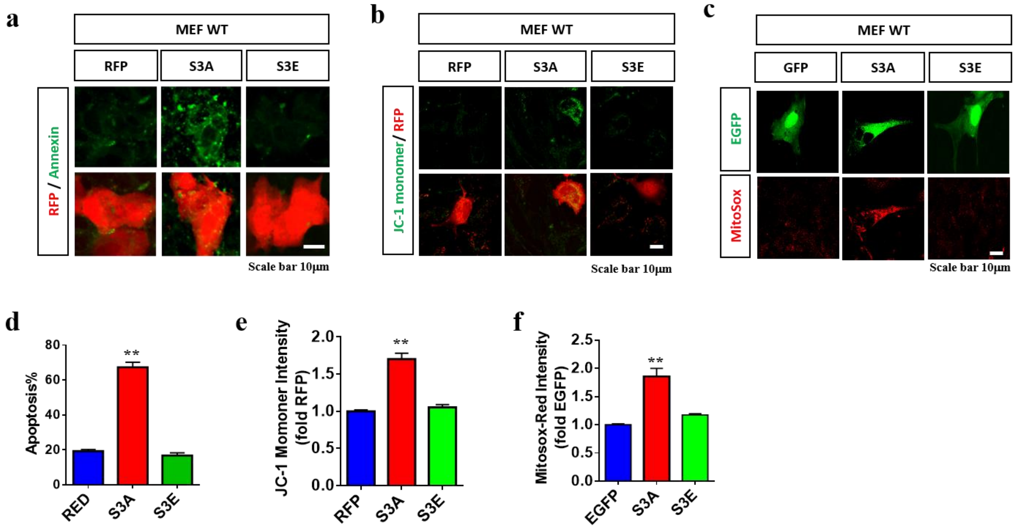

Supplemental Fig. 7

Fig.2 Uncropped western blots

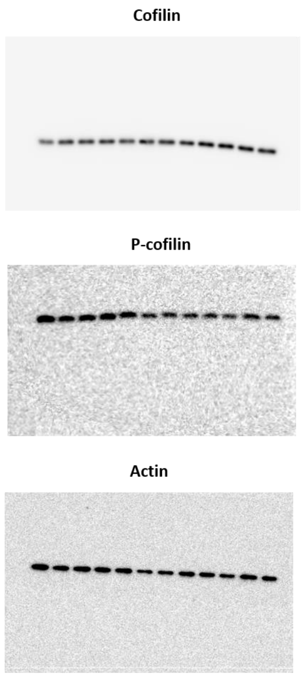

Fig.3 Uncropped western blots

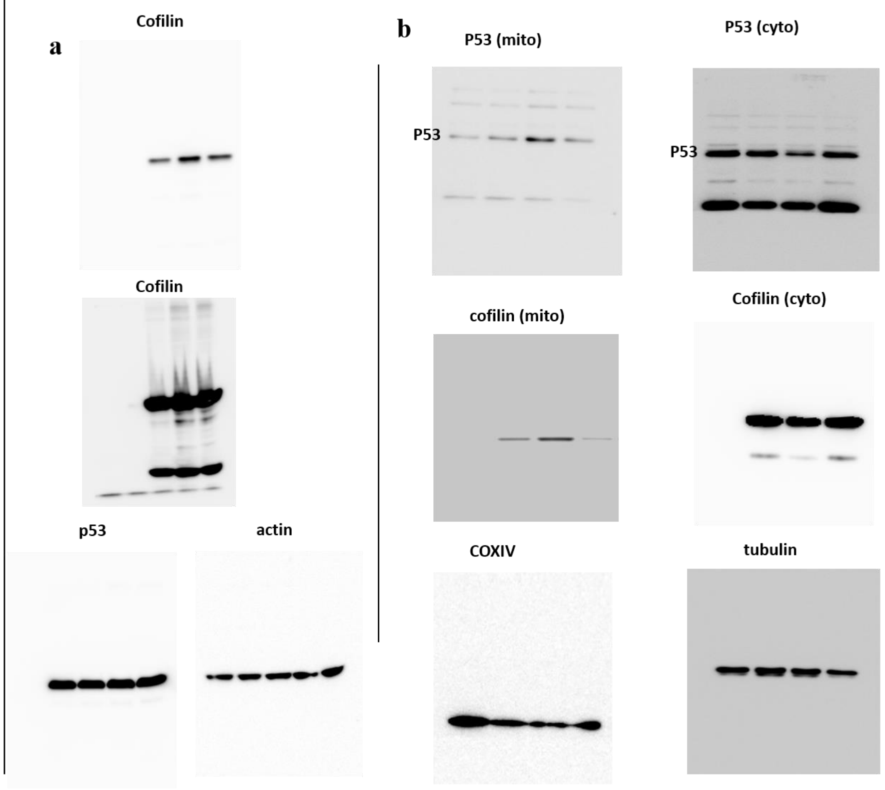

Supplemental Fig. 7 continued

Fig.3 Uncropped western blots

c

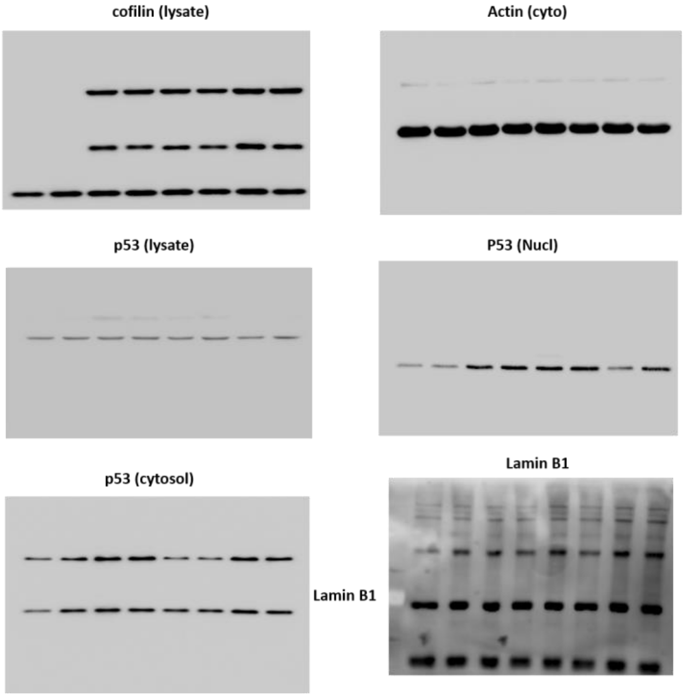

Fig.4 Uncropped western blots

e

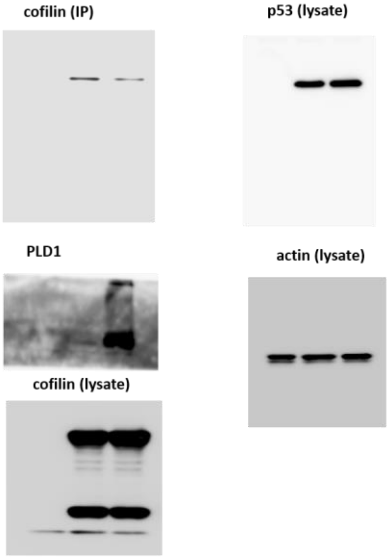

Fig.5 Uncropped western blots

a

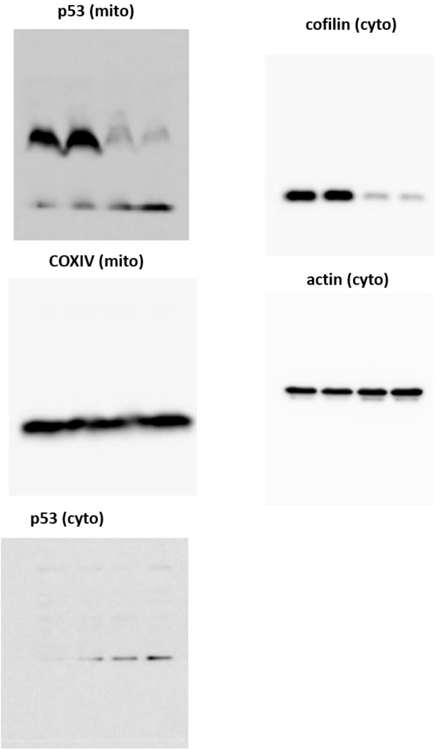

Supplemental Fig. 7 continued

c

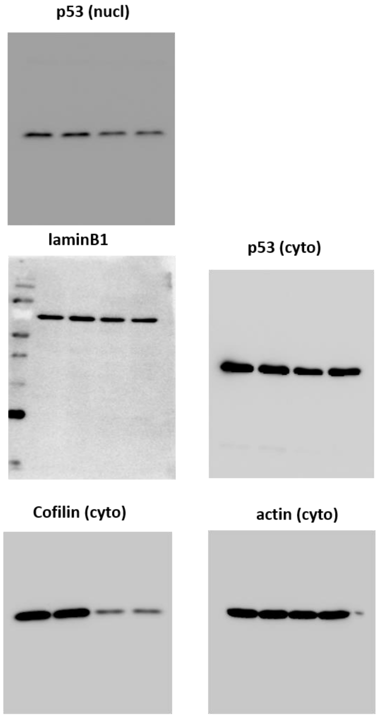

Supplemental Fig. 7 continued

Fig.6 Uncropped western blots

a

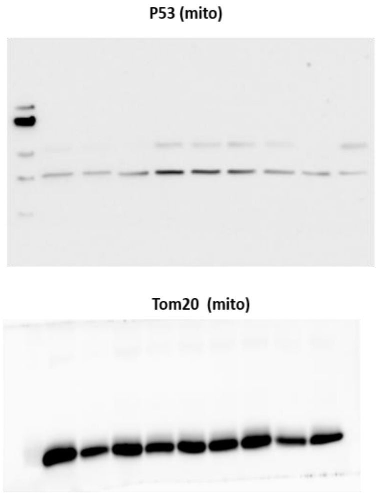

e

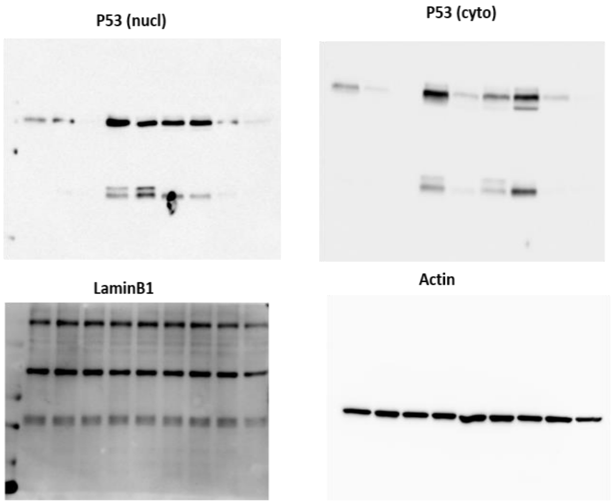

Supplement: Supplementary file 1 — Supplementary Information [file 41598_2017_9996_MOESM1_ESM.pdf]
